# *In vitro* characterization of [^125^I]HY-3-24, a selective ligand for the dopamine D3 receptor

**DOI:** 10.3389/fnins.2024.1380009

**Published:** 2024-04-09

**Authors:** Ji Youn Lee, Ho Young Kim, Paul Martorano, Aladdin Riad, Michelle Taylor, Robert R. Luedtke, Robert H. Mach

**Affiliations:** ^1^Department of Radiology, Perelman School of Medicine, University of Pennsylvania, Philadelphia, PA, United States; ^2^Department of Pharmacology and Neuroscience, University of North Texas Health Science Center, Fort Worth, TX, United States

**Keywords:** [^**125**^I]HY-3-24, dopamine D3 receptor ligand, *in vitro* binding assay, islands of Calleja, nonhuman primates, autoradiography

## Abstract

**Introduction:**

Dopamine D3 receptor (D3R) ligands have been studied for the possible treatment of neurological and neuropsychiatric disorders. However, selective D3R radioligands for *in vitro* binding studies have been challenging to identify due to the high structural similarity between the D2R and D3R. In a prior study, we reported a new conformationally-flexible benzamide scaffold having a high affinity for D3R and excellent selectivity vs. D2R. In the current study, we characterized the *in vitro* binding properties of a new radioiodinated ligand, [^125^I]HY-3-24.

**Methods:**

*In vitro* binding studies were conducted in cell lines expressing D3 receptors, rat striatal homogenates, and rat and non-human primate (NHP) brain tissues to measure regional brain distribution of this radioligand.

**Results:**

HY-3-24 showed high potency at D3R (*K_i_* = 0.67 ± 0.11 nM, IC_50_ = 1.5 ± 0.58 nM) compared to other D2-like dopamine receptor subtypes (D2R *K_i_* = 86.7 ± 11.9 nM and D4R *K_i_* > 1,000). The *K_d_* (0.34 ± 0.22 nM) and B_max_ (38.91 ± 2.39 fmol/mg) values of [^125^I]HY-3-24 were determined. *In vitro* binding studies in rat striatal homogenates using selective D2R and D3R antagonists confirmed the D3R selectivity of [^125^I]HY-3-24. Autoradiography results demonstrated that [^125^I]HY-3-24 specifically binds to D3Rs in the nucleus accumbens, islands of Calleja, and caudate putamen in rat and NHP brain sections.

**Conclusion:**

These results suggest that [^125^I]HY-3-24 appears to be a novel radioligand that exhibits high affinity binding at D3R, with low binding to other D2-like dopamine receptors. It is anticipated that [^125^I]HY-3-24 can be used as the specific D3R radioligand.

## 1 Introduction

The dopamine D3 receptor (D3R) is an important receptor in the brain and is one of the members in D2-like receptor family, which also includes dopamine D2 (D2R) and dopamine D4 receptors (D4R). D3R is a G-protein coupled receptor (GPCRs) and inhibits cAMP signaling through Gα_i/o_ G-proteins (Robinson and Caron, 1996; Missale et al., 1998). D3R is predominantly located in limbic areas such as the ventral pallidum (Diaz et al., 1994, 1995), nucleus accumbens (NAc), olfactory tubercle (OT), and islands of Calleja (ICj), and has a lower density in the dorsal striatum in rat brain (Bouthenet et al., 1991; Levesque et al., 1992). The regional distribution of the D3R led to the conclusion that it may play an important role in neurological and neuropsychiatric disorders such as schizophrenia (Gurevich et al., 1997), Parkinson’s disease (Brooks, 2000; Elgueta et al., 2017), and drug addiction (Heidbreder et al., 2005; Sokoloff et al., 2006). Thus, targeting D3R has been pursued as a potential treatment of neuropsychiatric disorders, but the development of D3R ligands has faced numerous challenges over the past 2 decades.

As a member of the D2-like receptor family, the D3R has both structural and pharmacological similarities to the other members, especially the D2R (Missale et al., 1998). The D2R and D3R share 78% amino acid sequence homology in the transmembrane domains (TM) (Giros et al., 1990; Sokoloff et al., 1990) and follow the same signaling pathway (Robinson and Caron, 1996; Cho et al., 2008). Furthermore, there are brain regions that express both D2 and D3 receptors but with different receptor densities (Missale et al., 1998; Sun et al., 2012).

Several radioligands such as [^3^H]WC-10 (Xu et al., 2009, 2010), [^3^H]spiperone (Vile et al., 1995; Zhen et al., 2010), [^125^I]IABN (Luedtke et al., 2000), and [^125^I](R,S)-trans-7-OH-PIPAT (Burris et al., 1994; Stanwood et al., 2000) have been used in radioligand binding assays and autoradiography studies of the D3 receptor. However, all of these radioligands have an affinity for D2 receptors, which makes it difficult to measure the density of D3 receptors in tissues where both D2 and D3 receptors are present. For example, [^3^H]WC-10, the most D3-selective of the radioligands described above, requires a duo-radioligand study with [^3^H]raclopride and the use of simultaneous equations to calculate the fractional D2 and D3 occupancy of each radioligand to D2 and D3 receptors quantify D3 receptor density in tissue sections (Xu et al., 2009). Therefore, there is a need to develop a highly D3 selective radioligand that is capable of quantifying D3 receptor density without the need of complex calculations.

**SCHEME 1:**
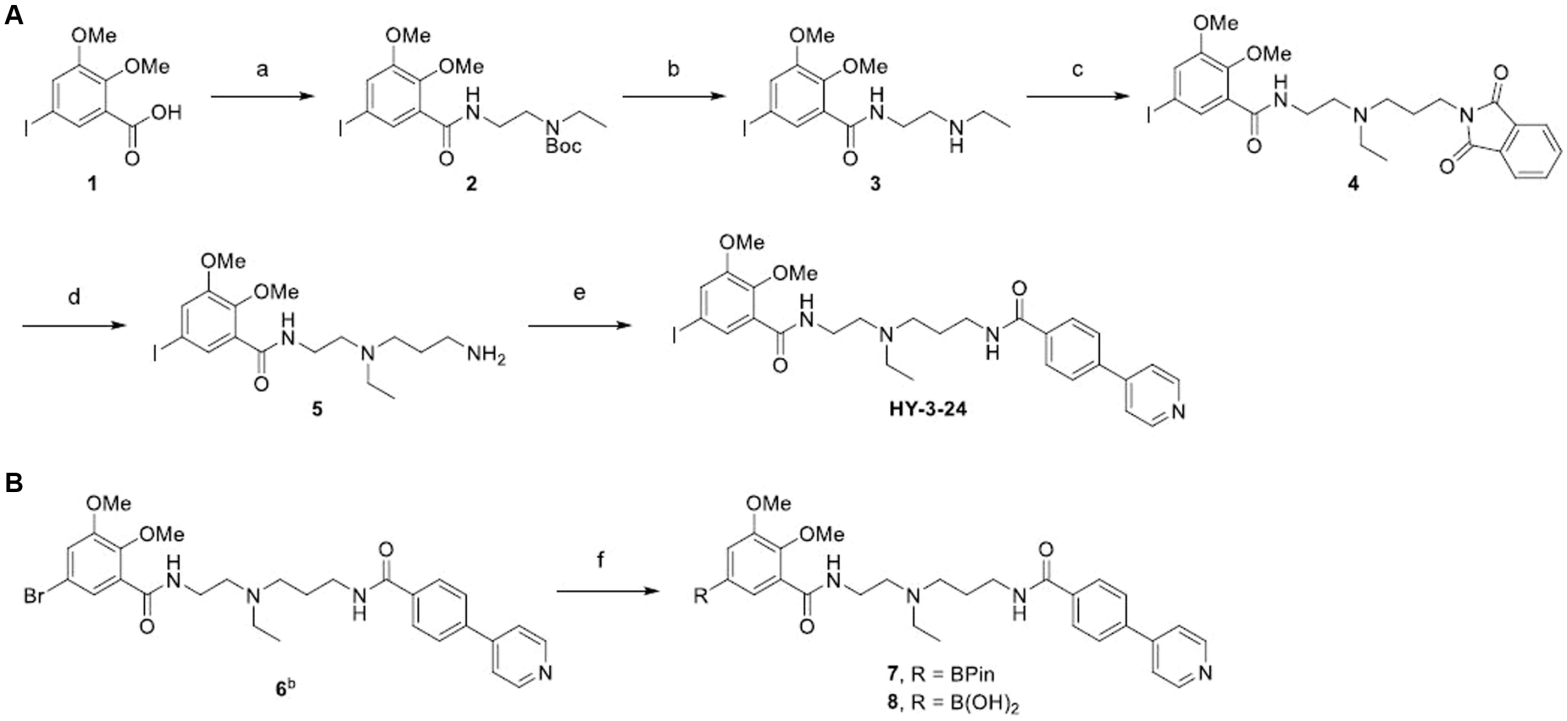
^a^Synthesis of standard **(A)** and precursors **(B)**. ^a^Reagents and conditions: (a) *tert*-butyl (2-aminoethyl)ethylcarbamate, HBTU, DIPEA, DMF, RT, 24 h; (b) TFA, CH_2_Cl_2_, 0°C to RT, 1 h; (c) *N*-(3-bromopropyl)phthalimide, K_2_CO_3_, DMF, 65°C, 16 h; (d) hydrazine hydrate, EtOH, 75°C, 3 h; (e) 4-(4-pyridyl)benzoic acid, SOCl_2_, 3 h, then, 5, CH_2_Cl_2_, RT, 1.5 h; (f) Pd(dppf)Cl_2_, bis(pinacolato)diboron, potassium acetate, 1,4-dioxane, 90°C, 3.5 h, ^b^Compound 6 was prepared according to the reported method (Kim et al., 2022).

Metoclopramide was developed as a dopamine receptor antagonist (Peringer et al., 1975; Elliott et al., 1978; Stanley and Wilk, 1979) and was approved by the Food and Drug Administration (FDA) for use for the treatment of gastroparesis. However, it has limitations for use in other therapeutic applications such as neurological and neuropsychiatric disorders because of its low affinity and selectivity D2 and D3 receptors (*K_i_* = 21 ± 1 nM for hD2R and *K_i_* = 27 ± 3 nM hD3R; Yang et al., 2000). To address these limitations, we designed a new scaffold based on metoclopramide in which an arylalkyl moiety was introduced to enable an interaction with the secondary binding site of the D3R (Kim et al., 2023). This new scaffold was found to have a high affinity for the D3R and excellent selectivity vs. the D2R. The structure–activity relationship study also identified the bromo analog (compound 6 in Scheme 1) as having a high affinity and selectivity for the D3R (*K_i_* = 107 ± 5 nM for hD2R and *K_i_* = 1.1 ± 0.1 nM hD3R). In the current study, we describe the properties of a new radioligand, [^125^I]HY-3-24 that possesses an enhanced affinity for targeting D3R, an increased D3R selectivity vs. D2R, and low off-target binding to other CNS targets. The pharmacological profile of this new radioligand was characterized by *in vitro* binding studies in tissue homogenates and tissue sections. Our results show that [^125^I]HY-3-24 is a novel radioligand for use in *in vitro* binding and autoradiography studies of the D3R.

## 2 Materials and methods

### 2.1 Chemistry

#### 2.1.1 General

5-Iodo-2,3-dimethoxybenzoic acid (1) was synthesized via electrophilic iodination using 5-Iodo-2,3-dimethoxybenzoic (Joshua et al., 2008) and *tert*-butyl (2-aminoethyl)ethylcarbamate were synthesized according to the known method (Komiya et al., 2016). ^1^H and ^13^C NMR spectra were obtained on a Bruker NEO-400 spectrometer. Chemical shifts (δ) were recorded in parts per million (ppm) relative to the deuterated methanol as an internal reference. Mass spectra (*m/z*) were recorded on a 2695 Alliance LC-MS using positive electrospray ionization (ESI^+^).

##### 2.1.1.1 *tert*-Butyl ethyl(2-(5-iodo-2,3-dimethoxybenzamido)ethyl)carbamate (2)

In a mixture of 1 (2.4 g, 7.8 mmol), *tert*-butyl (2-aminoethyl)ethylcarbamate (2.2 g, 11.7 mmol), and HBTU (4.5 g, 11.7 mmol) in 26 mL of DMF, DIPEA (2 mL, 11.7 mmol) was slowly added. The mixture was stirred at room temperature for 24 h and diluted with ethyl acetate. The organic layer was washed with water and brine, dried over anhydrous sodium sulfate, filtered and concentrated *in vacuo*. The crude product was purified by flash chromatography on silica gel (EtOAc/*n*-hexane = 1:4) to afford 2 (3.6 g, 96% yield) as a colorless oil. (^1^H NMR, 400 MHz, MeOD): δ = 7.66 (s, 1H), 7.41 (s, 1H), 3.86 (s, 6H), 3.52 (t, *J* = 5.4 Hz, 2H), 3.43 (t, *J* = 6.0 Hz, 2H), 3.30 (q, *J* = 6.9 Hz, 2H), 1.42 (s, 9H), 1.12 (t, *J* = 7.0 Hz, 3H); (^13^C NMR, 100 MHz, MeOD): δ = 167.0, 155.1, 148.9, 131.5, 125.5, 87.6, 62.1, 57.1, 39.6, and 28.9; ESI-MS *m/z* calculated for C_18_H_28_IN_2_O_5_^+^ [M + H]^+^ 479.3; found 479.4.

##### 2.1.1.2 *N*-(2-(Ethylamino)ethyl)-5-iodo-2,3-dimethoxybenzamide (3)

A solution of 2 (3.6 g, 7.5 mmol) in 20 mL of CH_2_Cl_2_ was placed in an ice bath. 20 mL of TFA was slowly added. The mixture was warmed to room temperature and stirred for 1 h. After the reaction was completed by TLC, the volatiles were removed under the reduced pressure. The crude product was purified by flash chromatography on silica gel (CH_2_Cl_2_/7 N NH_3_ in MeOH = 20:1) to afford 3 (1.9 g, 66% yield) as a colorless oil. (^1^H NMR, 400 MHz, MeOD): δ = 7.62 (*d*, *J* = 2.0 Hz, 1H), 7.41 (*d*, *J* = 1.9 Hz, 1H), 3.87 (s, 3H), 3.86 (s, 3H), 3.50 (t, *J* = 6.4 Hz, 2H), 2.80 (t, *J* = 6.4 Hz, 2H), 2.67 (*q*, *J* = 7.2 Hz, 2H), 1.13 (t, *J* = 7.2 Hz, 3H); (^13^C NMR, 100 MHz, MeOD): δ = 167.2, 155.1, 148.7, 131.3_1_, 131.2_9_, 125.3, 87.6, 62.0, 57.1, 49.4, 44.7, 40.5, 15.1; ESI-MS *m/z* calculated for C_13_H_21_IN_2_O_3_^+^ [M + 2H]^+^ 380.2; found 380.3.

##### 2.1.1.3 *N*-(2-((3-(1,3-Dioxoisoindolin-2-yl)propyl)(ethyl)amino)ethyl)-5-iodo-2,3-dimethoxybenzamide (4)

To a mixture of 3 (1.8 g, 4.7 mmol) and *N*-(3-bromopropyl)phthalimide (2.5 g, 9.4 mmol) in 16 mL of DMF, K_2_CO_3_ (1.6 g, 11.7 mmol) was added. The mixture was heated at 65°C for 16 h and cooled to room temperature. Another CH_2_Cl_2_ was added and the organic later was washed with aqueous saturated NaHCO_3_ solution, dried over anhydrous sodium sulfate and filtered. The solvent was removed under reduced pressure and the crude product was purified by flash chromatography on silica gel (CH_2_Cl_2_/MeOH = 20:1) to afford 4 (2 g, 76% yield) as a yellow oil. (^1^H NMR, 400 MHz, CDCl_3_): δ = 7.90–7.74 (m, 4H), 7.67 (*d*, *J* = 1.8 Hz, 1H), 7.37 (*d*, *J* = 1.6 Hz, 1H), 3.86 (s, 3H), 3.84 (s, 3H), 3.71 (t, *J* = 7.1 Hz, 2H), 3.45 (t, *J* = 6.4 Hz, 2H), 2.68–2.56 (m, 6H), 1.90–1.82 (m, 2H), 1.03 (t, *J* = 7.1 Hz, 3H); (^13^C NMR, 100 MHz, MeOD): δ = 170.0, 166.5, 155.1, 149.1, 135.4, 133.5, 131.7, 130.3, 125.5, 124.2, 87.6, 62.1, 60.7, 57.1, 53.2, 52.0, 38.8, 37.3, 36.3, 32.6, 27.3, 11.8; ESI-MS *m/z* calculated for C_24_H_30_IN_3_O_5_^+^ [M + 2H]^+^ 567.4; found 567.6.

##### 2.1.1.4 *N*-(2-((3-Aminopropyl)(ethyl)amino)ethyl)-5-iodo-2,3-dimethoxybenzamide (5)

To a solution of 4 (2 g, 3.5 mmol) in 35 mL of EtOH, hydrazine hydrate (0.54 mL, 10.6 mmol) was added. The mixture was heated at 75°C for 3 h and cooled to room temperature. NaOH was added till the crude product was extracted to CH_2_Cl_2_. The organic layer was dried over anhydrous sodium sulfate, filtered and concentrated *in vacuo*. The crude product was purified by flash chromatography on silica gel (CH_2_Cl_2_/7 N NH_3_ in MeOH = 10:1) to afford 5 (1.2 g, 73% yield) as a yellow oil. (^1^H NMR, 400 MHz, MeOD): δ = 7.71 (*d*, *J* = 2.0 Hz, 1H), 7.43 (*d*, *J* = 2.0 Hz, 1H), 3.88 (s, 3H), 3.87 (s, 3H), 3.47 (t, *J* = 6.6 Hz, 2H), 2.67 (t, *J* = 6.8 Hz, 4H), 2.62 (*q*, *J* = 7.3 Hz, 2H), 2.56 (t, *J* = 7.2 Hz, 2H), 1.69–1.62 (m, 2H), 1.07 (t, *J* = 7.1 Hz, 3H); (^13^C NMR, 100 MHz, MeOD): δ = 166.5, 155.2, 149.1, 131.7, 130.3, 125.6, 87.7, 62.1, 57.1, 53.2, 52.4, 48.6, 41.1, 38.7, 31.0, 12.0; ESI-MS *m/z* calculated for C_16_H_28_IN_3_O_3_^+^ [M + 2H]^+^ 437.3; found 437.3.

##### 2.1.1.5 *N*-(2-(Ethyl(3-(4-(pyridin-4-yl)benzamido)propyl)amino)ethyl)-5-iodo-2,3-dimethoxybenzamide (HY-3-24)

To a vial containing 4-(4-pyridyl)benzoic acid (24 mg, 0.12 mmol), thionyl chloride (0.26 mL, 3.6 mmol) was added. The mixture was stirred for 3 h followed by the volatiles were removed under reduced pressure. To the residue, a solution of 5 (44 mg, 0.1 mmol) in 2 mL of CH_2_Cl_2_ and Et_3_N (35 μL, 0.25 mmol) were added in a sequence. The mixture was stirred for 1.5 h and concentrated *in vacuo*. The crude product was purified by flash chromatography on silica gel (CH_2_Cl_2_/7 N NH_3_ in MeOH = 40:1) to afford 6 (45 mg, 73% yield) as a colorless oil. (^1^H NMR, 400 MHz, MeOD): δ = 8.61 (*d*, *J* = 5.1 Hz, 2H), 7.91 (*d*, *J* = 8.0 Hz, 2H), 7.80 (*d*, *J* = 8.1 Hz, 2H), 7.73 (*d*, *J* = 5.1 Hz, 2H), 7.68 (s, 1H), 7.37 (s, 1H), 3.86 (s, 3H), 3.82 (s, 3H), 3.48 (m, 4H), 2.70 (t, *J* = 6.3 Hz, 2H), 2.64 (t, *J* = 7.1 Hz, 4H), 1.87–1.80 (m, 2H), 1.08 (t, *J* = 7.1 Hz, 3H); (^13^C NMR, 100 MHz, MeOD): δ = 169.4, 166.5, 155.1, 150.9, 149.6, 149.1, 141.8, 136.6, 131.7, 130.2, 129.3, 128.4, 125.6, 123.4, 87.7, 62.1, 57.0, 55.0, 53.4, 52.3, 48.7, 39.8, 38.8, 27.8, 11.9; ESI-MS *m/z* calculated for C_28_H_34_IN_4_O_4_^+^ [M + H]^+^ 617.5; found 617.4 HRMS (ESI) for C_28_H_33_IN_4_NaO_4_^+^ [M + Na]^+^ 639.1444; found 639.1439.

##### 2.1.1.6 *N*-(2-(Ethyl(3-(4-(pyridin-4-yl)benzamido)propyl)amino)ethyl)-2,3-dimethoxy-5-(4,4,5,5-tetramethyl-1,3,2-dioxaborolan-2-yl)benzamide (7)

To a solution of 6 (150 mg, 0.26 mmol) in 2 mL of 1,4-dioxane, bis(pinacolato)diboron (200 mg, 0.79 mmol), Pd(dppf)Cl_2_ (19 mg, 0.026 mmol), and potassium acetate (153 mg, 1.56 mmol) were added. The mixture was heated 90°C for 3.5 h and cooled to room temperature. After checked that 6 was used up by LC–MS, the mixture was diluted with EtOAc. The organic layer was washed with water and brine, dried over sodium sulfate and filtered. The solvent was removed under reduced pressure and the crude product was purified by flash chromatography on silica gel (CH_2_Cl_2_/MeOH = 10:1) to afford 7 (34 mg, 21% yield) as a colorless oil. (^1^H NMR, 400 MHz, MeOD): δ = 8.62 (*d*, *J* = 5.1 Hz, 2H), 7.92 (*d*, *J* = 8.3 Hz, 2H), 7.82 (*d*, *J* = 8.3 Hz, 3H), 7.74 (*d*, *J* = 6.0 Hz, 2H), 7.44 (s, 1H), 3.92 (s, 3H), 3.87 (s, 3H), 3.57 (t, *J* = 6.3 Hz, 2H), 3.48 (t, *J* = 6.7 Hz, 2H), 2.85 (t, *J* = 6.1 Hz, 2H), 2.82–2.76 (m, 4H), 1.93–1.86 (m, 2H), 1.32 (s, 12H), 1.14 (t, *J* = 7.2 Hz, 3H); (^13^C NMR, 100 MHz, MeOD): δ = 169.7, 168.7, 153.7, 151.6, 150.9, 149.7, 142.0, 136.5, 129.9, 129.3, 128.4, 128.2, 123.4, 122.0, 85.5, 76.0, 62.1, 56.6, 54.9, 53.6, 52.2, 39.4, 38.3, 27.4, 25.3, 25.2, 11.3; ESI-MS *m/z* calculated for C_34_H_46_BN_4_O_6_^+^ [M + H]^+^ 617.6; found 617.6 HRMS (ESI) for C_34_H_46_BN_4_O_6_^+^ [M + H]^+^ 617.3510; found 617.3530.

##### 2.1.1.7 (3-((2-(Ethyl(3-(4-(pyridin-4-yl)benzamido)propyl)amino)ethyl)carbamoyl)-4,5-dimethoxyphenyl)boron-ic acid (8)

8 was synthesized using 6 (377 mg, 0.66 mmol) in the same condition as 7 and the crude product was purified by preparative HPLC [stationary phase: luna® 5 μm C18(2), 100 Å, 21.2 mm × 150 mm, mobile phase: 100% of 0.1% TFA in water for 6 min, 100 to 0% of 0.1% TFA in water/MeCN for 16 min and 100% for 5 min, wavelength 254 nm, flow rate: 21.2 mL/min, retention time: 11.9 min]. The solvent was removed by liophilization and the desired compound 8 (63 mg, 0.12 mmol) was obtained as a white solid. (^1^H NMR, 400 MHz, MeOD): δ = 8.86 (*d*, *J* = 6.8 Hz, 2H), 8.31 (*d*, *J* = 6.8 Hz, 2H), 8.05–8.00 (m, 4H), 7.80 (br s, 1H), 7.51 (br s, 1H), 3.93 (s, 3H), 3.88 (s, 3H), 3.83 (t, *J* = 6.0 Hz, 2H), 3.55 (t, *J* = 6.4 Hz, 2H), 3.47–3.37 (m, 6H), 2.16–2.09 (m, 2H), 1.38 (t, *J* = 7.2 Hz, 3H); (^13^C NMR, 100 MHz, MeOD): δ = 170.3, 169.7, 156.9, 153.5, 144.5, 139.5, 137.8, 129.8, 129.3, 127.1, 125.6, 122.3, 62.0, 56.7, 53.7, 49.8, 38.0, 36.5, 25.7, 9.1; ESI-MS *m/z* calculated for C_28_H_35_BN_4_O_6_^+^ [M + H]^+^ 535.4; found 535.5 HRMS (ESI) for C_28_H_35_BN_4_O_6_^+^ [M + H]^+^ 535.2728; found 535.2738.

### 2.2 Radioligand binding assays

#### 2.2.1 D2R/D3R/D4R binding assay

The dopamine receptor binding assay was performed according to previous studies (Luedtke et al., 2000; Vangveravong et al., 2006; Reilly et al., 2017), and human D2R or D3R or D4 transfected HEK293 cells were used for dopamine receptor binding assay. The cells were grown in Dulbecco’s Modified Eagle Medium including 10% FBS, 0.1% Penn Strep, 50 mg/mL of geneticin (G418) for human D2R or D3R transfected HEK293 cells, and 10 mg/mL of puromycin for human D4 HEK293 cells at 5% CO_2_, 37°C. The cells were collected by centrifuge (6,000 × *g*, 10 min), and the supernatant was discarded. Cold homogenization buffer (50 mM Na Hepes, 0.1 mM EDTA, 1 mM DTT, pH 7.4) was added into the pellet and homogenized by a polytron (Brinkmann Instruments, Westbury, NY, United States), and centrifuged at 40,000 × *g* for 10 min. This process was repeated two more times to get final pellet, and the membranes were then resuspended with HB buffer including 5 mM MgCl_2_ and stored at −80°C until use.

HY-3-24 was prepared in 10^−5^ to 10^−11^ M concentration range with assay buffer (50 mM Tris–HCl, 150 mM NaCl, 10 mM EDTA, pH 7.5). Membrane homogenates (5–15 μg) and [^125^I]IABN (approximately 0.5 nM) was added into serial dilutions of the compound and incubated at 37°C, for 60 min. Total binding volume was 150 μL, and 20 μM of (+)-butaclamol was used to define non-specific binding. Cold buffer (10 mM Tris–HCl, 150 mM NaCl, pH 7.5) was added into the reaction tube to terminate binding assay, and mixture was quickly filtered through a Schleicher and Schuell No.32 filter (GE Heathcare Bio-Sciences, Pittsburgh, PA, United States). The membrane was washed with cold buffer (three times), and collected filters were counted on a gamma counter (GMI, Minnesota, United States). The results were analyzed by nonlinear regression, and the data were reported mean ± SEM values by three independent experiments.

#### 2.2.2 Sigma receptors binding assay

The preparation of protein homogenates and sigma binding assay were conducted as described previously (Luedtke et al., 2000). For sigma-1 binding assay, [^3^H]-(+)-pentazocine (50 μL, ~50,000 cpm) and HY-3-24 (concentrations ranging from 10^−5^ to 10^−11^ M) were mixed, guinea pig brain homogenates (100 μg/100 μL) were added and the mixture was incubated at 5% CO_2_, 37°C for 90 min. The assay was terminated by adding cold buffer (10 mM Tris–HCl, 150 mM NaCl, pH 7.5), and the mixture was filtered htrough Whatman *CF*/C filters (Brandel Inc., Maryland, United States) which were soaked in 1% polyethyleneimine (PEI). The collected filter was washed three times using cold buffer; non-specific binding was determined in the presence of 10 μM of haloperidol. The collected filters were mixed with 3 mL of microscint™20 by and counted overnight (1 min/well) using a beta counter (MicroBeta^2^®, PerkinElmer, United Kingdom). The data were analyzed using PRISM 9.

For sigma-2 binding assay, HY-3-24 prepared in 10^−5^ to 10^−11^ M concentration range was mixed with [^125^I]RHM4 (50 μL,~200,000 cpm) and rat liver homogenates (15 μg/100 μL). The mixture was vortexed briefly and incubated at room temperature (RT) for 90 min. The [^125^I]RHM4-bound membrane was collected through Whatman *CF*/C filters by 24-well harvester (Brandel Inc., Maryland, United States), and washed with 4 mL of cold buffer. The filters were counted on a gamma counter (PerkinElmer, Massachusetts, United States). Cold RHM4 was used for defining non-specific binding. The *K_i_* value was obtained by PRISM 9.

#### 2.2.3 *β*-arrestin recruitment assay for D3R

Chinese hamster ovary CHO-K1 cells (CHO-K1) which were over expressed human D3R were cultured in assaycomplete™ cell culture kit 107 (DiscoverX, Fremont, CA, United States). Cells were seeded at a density of 25,000 cells per well of 96-well plate, and incubated at 5% CO_2_, 37°C. After 46 h, HY-3-24 was (10^−5^ to 10^−11^) in phosphate-buffered saline (PBS) was added to the cells. The plates were incubated for 30 min at 5% CO_2_, 37°C for the agonist binding assay; for the antagonist assay, cells were treated with 30 nM (EC_80_) of dopamine and then the mixture was incubated for 90 min. PathHunter™ *β*-arrestin recruitment assay kit (DiscoverX, Fremont, CA, United States) was added to each well, and the plate was incubated for 80 min at RT under the dark condition. The chemiluminescent signal was measured on a PerkinElmer Enspire plate reader (PerkinElmer, Boston, MA, United States). Data were analyzed by Prism followed by non-linear regression.

### 2.3 Radiochemistry

Test labeling was performed using two different precursors 7 or 8. First, 100 μL of 100 mM of Cu(pyridine)_4_(OTf)_2_ in MeCN solution and 100 μL of 100 mM of 3,4,7,8-tetramethyl-1,10-phenanthroline in MeOH solution were mixed to make 50 mM catalyst mixture. To a solution of 7 or 8 (100 μg, 0.2 μmol) in 50 μL of MeOH, 100 μL of 50 mM catalyst mixture was added followed by 2 μL of Na[^125^I] (~ 2.22 MBq). The reaction mixture was incubated at room temperature for 30 min or heated at 100°C for 10 min. 300 μL of 0.1 M ammonium acetate buffer (pH 4.6) was added for quenching, and each reaction was assessed by HPLC [stationary phase: Luna® 5 μm C18 100 Å, 10 mm × 250 mm, mobile phase: 68% 0.1 M ammonium acetate buffer (pH 4.6) in MeCN, wavelength 254 nm, flow rate: 4 mL/min, retention time: 8.6 min]. For scale-up radiolabeling studies, 8 was used as the precursor. To a solution of 8 (200 μg, 0.4 μmol) in 50 μL of MeOH, 100 μL of 50 mM catalyst mixture and 100 μL of Na[^125^I] (318 MBq) in 0.1 N aq NaOH solution were added. The reaction mixture was heated at 100°C for 10 min and incubated at room temperature for 30 min. After the completion of the reaction, the mixture was quenched by 0.6 mL of aqueous mobile phase and the crude product was purified by preparative HPLC (stationary phase: Luna® 5 μm C18 100 Å, 10 mm × 250 mm, mobile phase: 75% 0.1 M ammonium acetate buffer in MeCN, wavelength 254 nm, flow rate: 4 mL/min, retention time: 22.5 min). The product fraction was collected in a tube containing 0.6 mL of ammonium hydroxide and 30 mL of water. The basic solution was passed on tC18 cartridge, and the cartridge was washed by 5 mL of water. The final product was eluted using 1 mL of ethanol. Radiochemical purity and molar activity was measured by analytical HPLC (stationary phase: Luna® 5 μm C18 100 Å, 10 mm × 250 mm, mobile phase: 68% 0.1 M ammonium acetate buffer in MeCN, wavelength 254 nm, flow rate: 4 mL/min, retention time: 8.4 min).

### 2.4 Distribution coefficient (Log*D*_7.4_)

In a separatory funnel, 10 mL of n-octanol and 20 mL of 0.01 M phosphate buffer (pH 7.4) solution were placed and 5 μL of [^125^I]HY-3-24 (700 kBq) was added. The mixture was vigorously shaken for 1 min followed by standing for 20 min to completely separate the layers. Five 1 mL of aliquots were taken from each phase and the radioactivity of each fraction was measured by a Wizard2 Automatic Gamma Counter. Log*D*_7.4_ was measured in quintuplicate and calculated as the logarithm of the ratio of radioactivity in organic phase to that in water phase.

### 2.5 Specific binding of **[**^**125**^**I]HY-3-24** on rat ventral striatum

Frozen rat brains (Sprague–Dawley rat, Male, 6–8 weeks old) were obtained from Innovative research, Inc. (Novi, Michigan, United States). The ventral striatum was isolated by dissection on ice. Cold homogenization buffer (HB, 20 mM Tris–HCl, 5 mM MgCl_2_, 1 mM EDTA, pH7.5) was added and the tissue homogenized by mortar and pestle on ice. Homogenized tissue was centrifuged at 10,000 × *g*, 4°C for 30 min. Supernatant was removed and pellet was mixed with cold HB buffer by vortex. This process was repeated two times to get rat ventral striatal homogenates; and protein amount was determined using a BCA protein assay kit. Homogenized tissue homogenates were stored at −80°C until use.

To confirm saturation time of [^125^I]HY-3-24 with prepared membrane, [^125^I]HY-3-24 (~0.3 nM) was mixed with 100 μg of membrane and incubated at RT up to 2 h. [^125^I]HY-3-24 bound protein was collected by fast filtration methods (*CF*/C filter, Brandel Inc., Maryland, United States) and counted using gamma counter (PerkinElmer, Massachusetts, United States).

For saturation binding study, [^125^I]HY-3-24 (0.001–1.6 nM concentration range) was incubated with 100 μg/100 μL of homogenized protein at RT for 60 min. Non-specific binding was defined the presence of 10 μM (+)-butaclamol, and total reaction volume was 200 μL. The reaction was finished by adding cold buffer (10 mM Tris–HCl, 150 mM NaCl, pH 7.5), and [^125^I]HY-3-24 bound protein was filtered by 24-well harvester (Brandel Inc., Maryland, United States) through *CF*/C filter soaked with 1% PEI, and washed three times with 4 mL cold buffer. The collected filter was counted by gamma counter and data were analyzed by PRISM 9. The values of B_max_ and *K_d_* were obtained by three individual experiments.

### 2.6 Competition binding study of [^125^I]HY-3-24

PD128907, (+)-PHNO, quinpirole, SCH23390, raclopride, and eticlopride were prepared in concentrations ranging from 10^−5^ to 10^−11^ M for competition binding assay with [^125^I]HY-3-24. Test compounds were mixed with 0.2–0.3 nM of [^125^I]HY-3-24 and rat ventral striatal membranes (100 μg/100 μL) were added. 10 μM of (+)-butaclamol was used for determination of non-specific binding. The final reaction volume was 200 μL and the assay mixture was incubated at RT for 60 min. [^125^I]HY-3-24 bound protein was filtered through *CF*/C filter by a Brandel 24-well harvester. The filter was washed with cold buffer (3 times) and counted by gamma counter. The value of *K_i_* was calculated using nonlinear regression analysis by PRISM 9 and the results was presented mean ± SD by three individual experiments.

For the D3R and D2R blocking studies, [^125^I]HY-3-24 (0.3 nM) was mixed with 100 μg of protein and incubated at RT for 2 h in the presence or absence of 30 nM cold HY-3-24, 100 nM SB-277-011A, 10 nM SB-277-011A, or 2.4 nM L-741,626. [^125^I]HY-3-24 bound protein was collected by fast filtration methods (GF/C filter soaked in 1% PEI, Brandel Inc., Maryland, United States) and counted using gamma counter (PerkinElmer, Massachusetts, United States).

### 2.7 *In vitro* autoradiography

Regional brain distribution of [^125^I]HY-3-24 binding was determined by *in vitro* autoradiography using Sprague–Dawley rat and non-human primate brains. A Cryotome (Leica Biosystems, Germany) was used for sectioning frozen brains by coronal and/or sagittal direction, and the thickness of tissue was 10–20 μm. Sectioned slides were stored at −80°C until use. The adjacent brain slides were warmed to RT for 20 min, the slides were incubated with cold reaction buffer (50 mM Tris–HCl, 150 mM NaCl, 1 mM EDTA, pH 7.5) at RT for 10 min, and the buffer was discarded. The slides were then incubated with 0.10.3 nM of [^125^I]HY-3-24 at RT for 1 h. For non-specific binding, 2 μM of (+)-butaclamol was used. After incubation, the slides were washed with cold buffer (three times) for 2 min, then air dried at RT for 10 min. Radiolabeled brain tissues were exposed to BAS imaging plate (Fujifilm, Tokyo, Japan) for 3 days, and the result was digitized by Typhoon FLA 7000 (GE Healthcare, Illinois, United States) and analyzed by Multi Gauge V3.0. For blocking studies comparing (+)-butaclamol and raclopride, brain slides were initially pretreated with blocking agent (50 nM) for 1 h. prior to the addition of the radioligand (0.3 nM).

## 3 Results

### 3.1 Chemistry

HY-3-24 and its boron-containing precursors for [^125^I]iodine labeling were synthesized as shown in Scheme 1. 5-Iodo-2,3-dimethoxybenzoic acid (1) was conjugated with a *Boc*-protected pendant amine to give 2; the *Boc* was removed using TFA to introduce the propyl linker in 63% yield for two steps (Kim et al., 2023). The linker was introduced in a good yield (76%) by *N*-alkylation of 3 with *N*-(3-bromopropyl)phthalimide. The phthalimide 4 was converted to the free amine 5 using hydrazine. After preparing 4-(pyridine-4-yl)benzoyl chloride *in situ*, a solution of 5 in CH_2_Cl_2_ was added with a base as an acid scavenger. HY-3-24 was successfully synthesized with a satisfactory yield (73%).

Two different precursors, pinacol boronic ester 7 and boronic acid 8 were synthesized to evaluate the optimal conditions for radioiodine labeling. For the insertion of boron, Miyaura borylation was performed using bromine functionalized substrate (Kim et al., 2023). Although 7 and 8 could be synthesized under the same reaction conditions, the purification procedure was different depending on the polarity of the prepared compound. 7 was purified by flash chromatography using a neutral CH_2_Cl_2_ and methanol mixture as a mobile phase, whereas 8 was prepared by HPLC purification using an aqueous 0.1% TFA in water and acetonitrile. It was found that the pinacol boronic ester was easily hydrolyzed on a silica material under an acidic conditions. 7 and 8 were prepared with yields of 21 and 18%, respectively.

### 3.2 *In vitro* receptor binding profiles and functional activity

Binding affinities for D2-like dopamine receptor subtypes were measured and the results are shown in Table 1 (Supplementary Figure S1). For the binding assay, transfected HEK293 cells expressing hD2/D3/D4R subtype were used; [^125^I]IABN was used as the radioligand. The *K_i_* value of HY-3-24 for D3R was 0.67 ± 0.11 nM, and the D2R *K_i_* value was 86.7 ± 11.9 nM (~129-fold selectivity for D3R). Furthermore, HY-3-24 was not active at D4R having a *K_i_* value 1,000 nM. Thus, HY-3-24 binds selectively to the D3R over the other D2-like dopamine receptor subtypes. Radioligand binding assays were also performed using HY-3-24 with [^3^H]pentazocine for sigma-1 and [^125^I]RHM4 for sigma-2. HY-3-24 did not bind to sigma-1 and sigma-2 receptors (Supplementary Figure S2).

**Table 1:** Pharmacological profiles of **HY-3-24**.

Radioligand binding assay	
Dopamine receptors	*K*_i_ (nM)
D2R	86.7 ± 11.9
D3R	0.67 ± 0.11
D4R	> 1,000
D2/D3 ratio	129
*β*-arrestin recruitment assay	
D3R	nM
EC_50_	N.A (not active)
IC_50_	1.5 ± 0.58

A *β*-arrestin recruitment assay was performed to assess D3R agonist activity and D3R antagonist activity. The EC_50_ of HY-3-24 was compared with dopamine for D3R agonist activity; there was no *β*-arrestin recruitment when the HY-3-24 was run in the agonist mode at a concentration ranging from 10^−5^ to 10^−11^ M (Table 1; Supplementary Figure S3A). When run in the antagonist mode, the IC_50_ value of HY-3-24 for the maximum inhibition of a dopamine (EC_80_ concentration) was 1.5 ± 0.58 nM (Table 1; Supplementary Figure S3B).

### 3.3 Comprehensive binding profiles for other aminergic GPCRs

The binding of HY-3-24 to various GPCRs was investigated through comprehensive screening by Psychoactive Drug Screening Program (PDSP) (Supplementary Table S1) (Besnard et al., 2012). These GPCRs included major neurotransmitter receptors family (e.g., dopamine, serotonin, histamine, opioid, muscarinic, adrenergic, sigma receptors, ɤ-amino-butyric acid type A, and benzodiazepine receptors) and transporters (e.g., dopamine, norepinephrine, and serotonin transporter). Consistent with the previous screening of 6 against these GPCRs (Kim et al., 2023), HY-3-24 had an overall low binding affinity to the GPCRs screened except 5-HT_2B,_ which moderate binding affinity (38 nM). Other receptors displaying modest affinity were 5-HT_3_ (904 nM), 5-HT_2A_ (310 nM), and moderate binding affinity at 5-HT_2C_ (613 nM).

### 3.4 Radiochemistry

**SCHEME 2:**
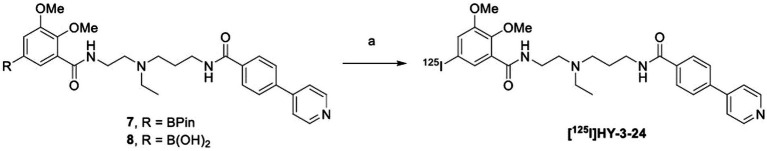
^a^Test labeling of [^125^I]HY-3-24 with different precursors. ^a^Reagents and conditions: (a) Na^125^I, Cu(py)_4_(OTf)_2_, 1,10-phenanthroline, MeOH/MeCN, RT for 30 min or 100°C for 10 min.

Cu-catalyzed [^125^I]iodination was performed based on the previously established method in our group (Scheme 2) (Reilly et al., 2018). Since both of pinacol boronic ester and boronic acid can be used for the precursor, the labeling was performed using 7 or 8 with different reaction conditions (Table 2). Test precursors 7 and 8 were successfully labeled with [^125^I]iodine in an excellent radiochemical yield (RCY) within a short time (Supplementary Figure S4). Particularly, the boronic acid 8 exhibited slightly higher RCY (97 or 94% at RT or 100°C, respectively) than the pinacol boronic ester 8 (89 or 88% at RT or 100°C, respectively). In the radio-HPLC, the impurity was confirmed unreacted [^125^I]iodine at RT and by-products at 100°C according to the retention time. When the reaction time was increased at 100°C, the by-products were significantly increased.

**Table 2:** Radiosynthesis conditions of **[**^**125**^**I]HY-3-24** using different precursors.

Precursor	*R*	Temp (°C)	Time (min)	RCY(%)^a^
7	BPin	RT	30	88.8 ± 2.3
7	BPin	100	10	88.3 ± 1.7
8	B(OH)_2_	RT	30	96.7 ± 0.5
8	B(OH)_2_	100	10	94.1 ± 1.2

^a^RCY (%) was calculated by radio-HPLC.

For the *in vitro* validation study, the boronic acid 8 was selected for the radiosynthesis of [^125^I]HY-3-24. The labeling conditions for the scale-up was optimized based on the test labeling results by heating at 100°C for 10 min and incubating at RT for 30 min. The amount of 8 was increased from 0.2 to 0.4 μmol. The RCY was 54.4 ± 10.1% (*n* = 4) that was calculated from the radioactivity of the isolated product and not decay corrected. The radiochemical purity of [^125^I]HY-3-24 was 99% and the molar activity was 1,400 Ci/mmol based on analytical HPLC (Supplementary Figure S5).

### 3.5 Determination of distribution coefficient (Log*D*_7.4_)

Log*D*_7.4_ of [^125^I]HY-3-24 was measured by the shake-flask method in quintuplicate and were determined as 2.41 ± 0.01. This Log*D*_7.4_ value demonstrated that [^125^I]HY-3-24 is well-balanced between solubility and permeability and suitable for drug candidate.

### 3.6 Pharmacological characterization of D3R binding sites in the ventral striatum

**Figure 1:**
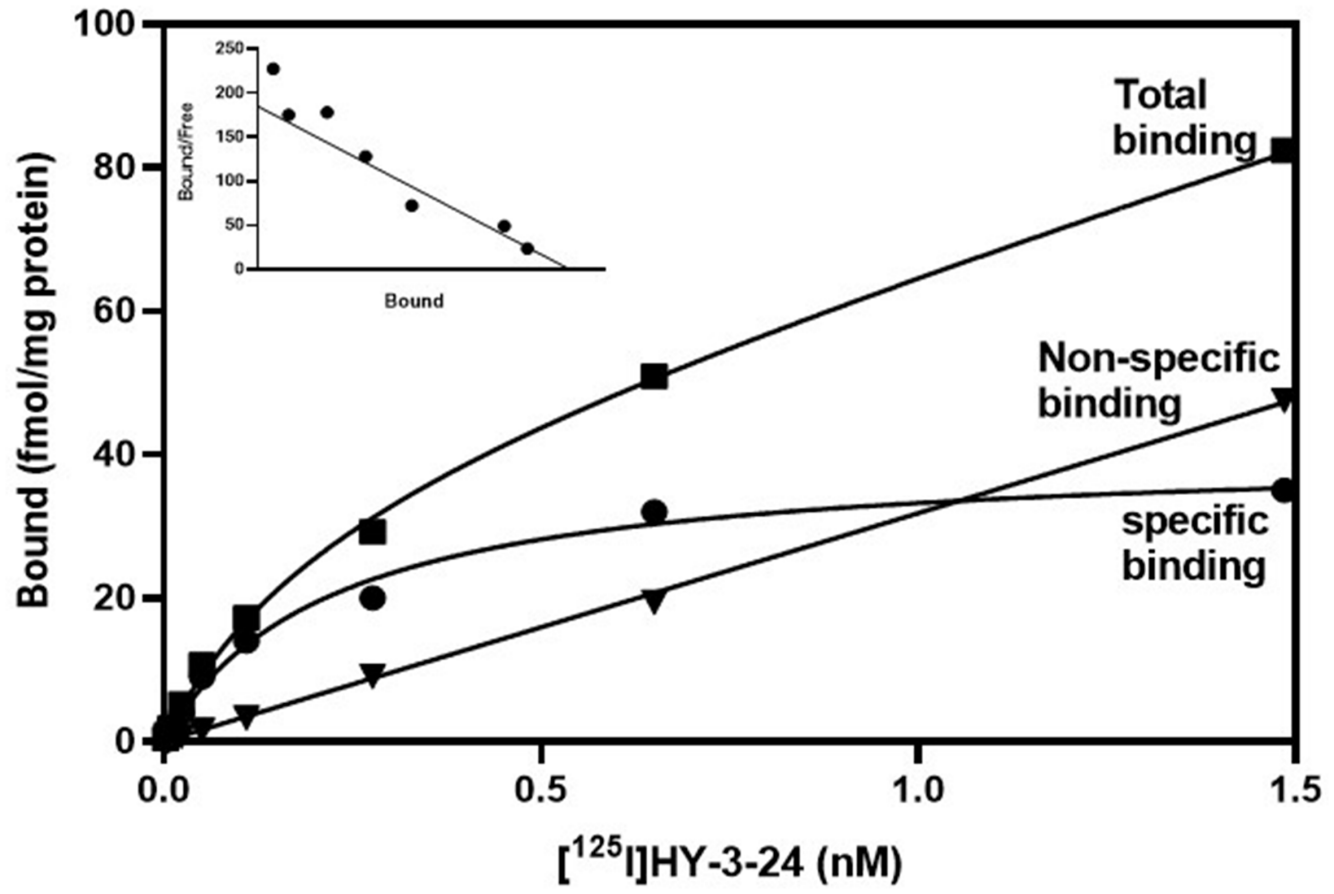
Saturation study of **[**^**125**^**I]HY-3-24** in rat ventral striatal membranes (nucleus accumbens and olfactory tubercle). Homogenized protein was incubated different concentrations of **[**^**125**^**I]HY-3-24** (0.001 ~ 1.6 nM range of concentration). Non-specific binding was determined the presence of 10 μM (+)-butaclamol. The *K*_d_ value (0.34 ± 0.22 nM) and B_max_ (38.91 ± 2.39 fmol/mg) were calculated by Prism 9. The data were obtained from three individual experiments. ■, total binding; ●, specific binding; ▼, non-specific binding.

To measure equilibrium property between [^125^I]HY-3-24 and D3R, [^125^I]HY-3-24 was incubated with 100 μg of protein at RT and the result was observed up to 2 h of incubation. Equilibrium was observed about 30 min after reaction, and it was persisted until 2 h (Supplementary Figure S6). The equilibrium conditions were used for direct binding studies. The percentage of specific binding was over 70%, and non-specific binding was less than 10% of total binding. Saturation curve were a single phase, and Scatchard plot was linear. Both results were indicated that [^125^I]HY-3-24 has a single binding site in rat ventral striatal membranes. The *K_d_* value for [^125^I]HY-3-24 was 0.34 ± 0.22 nM, and B_max_ was 38.91 ± 2.39 fmol/mg of protein (Figure 1). The *K_d_* value was calculated by Prism 9 and the data was obtained from three individual experiments.

**Figure 2:**
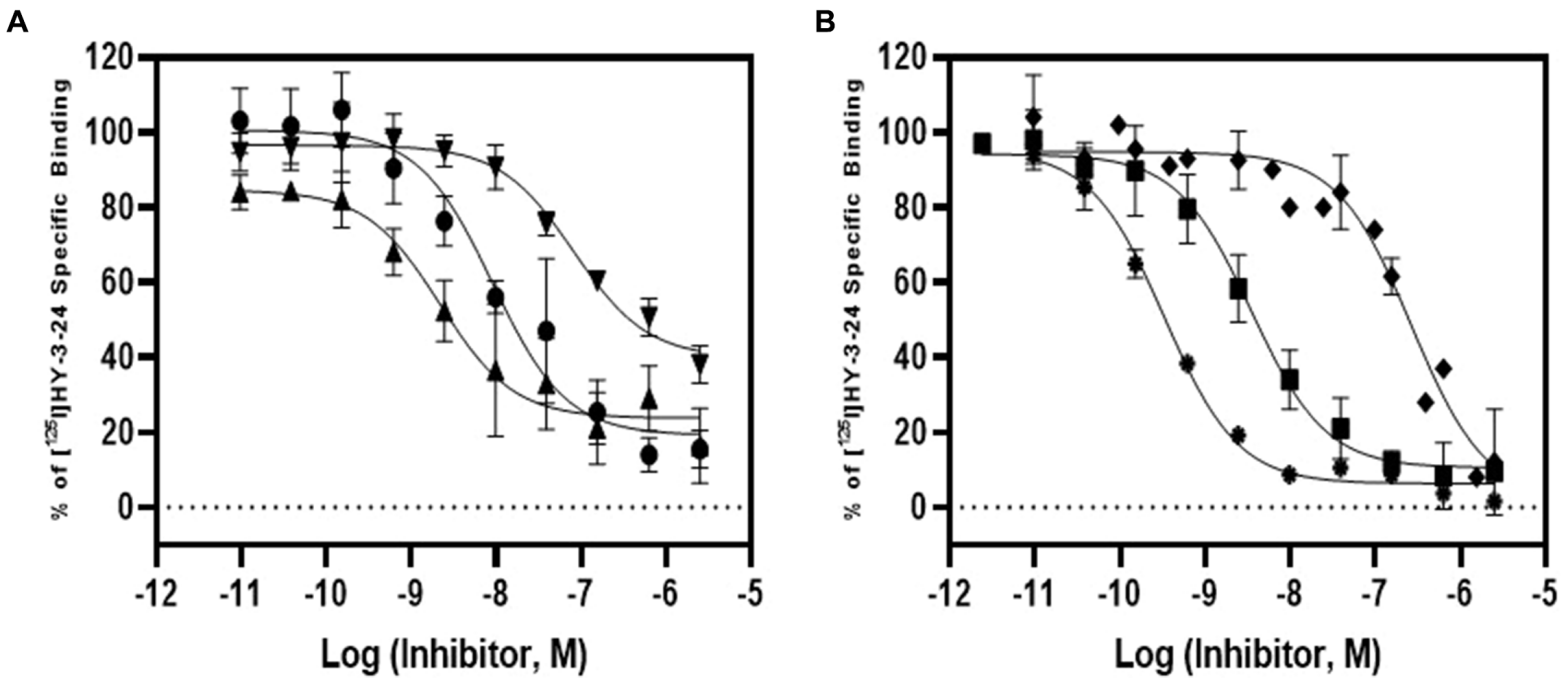
Competition study of [^125^I]HY-3-24 in rat ventral striatal membranes. Several dopamine agonists and antagonists (10^−12^ to 10^−4^) were incubated with ~0.3 nM of [^125^I]HY-3-24 and rat ventral striatal membrane. Data were analyzed and competition curves were generated by Prism 9. The results were obtained three individual experiments. **(A)** Agonist: ●, PD128907; ▲, (+)-PHNO; ▼, quinpirole; **(B)** antagonist: ♦, SCH23390; ■, raclopride; and ⁕, eticlopride.

The pharmacologic profile of [^125^I]HY-3-24 for D3R binding site was determined by comparison with reference compounds including dopamine agonists [PD128907, (+)-PHNO, and quinpirole] and antagonists (SCH23390, raclopride, and eticlopride) (Table 3; Figure 2). The binding study was conducted on rat ventral striatal homogenates, which highly express D3R. The *K_i_* values of dopamine ligands were: PD128907 (*K_i_* = 7.65 ± 1.15 nM), (+)-PHNO (*K_i_* = 1.72 ± 0.46 nM), quinpirole (*K_i_* = 63.8 ± 28.5 nM), SCH23390 (*K_i_* = 215 ± 61.7 nM), raclopride (*K_i_* = 2.51 ± 0.9 nM), and eticlopride (*K_i_* = 0.25 ± 0.02 nM). (+)-PHNO was the most potent agonist to inhibit the binding of [^125^I]HY-3-24; the rank order of the potency of the D3R agonists was: (+)-PHNO > PD128907 > > quinpirole. The most potent antagonist was eticlopride, and the rake order of potency for displacing [^125^I]HY-3-24 was: eticlopride > raclopride >> > SCH23390. Overall, the results for inhibiting the binding of [^125^I]HY-3-24 to D3R was consistent with the affinity of the agonist / antagonist for the D3R.

**Table 3:** Pharmacology of **[**^**125**^**I]HY-3-24** binding to homogenized protein of rat ventral striatum.

Compounds	*K*_i_ (nM)
Dopamine agonist	
PD128907	7.65 ± 1.15
(+)-PHNO	1.72 ± 0.46
Quinpirole	63.8 ± 28.5
Dopamine antagonist	
SCH23390	215 ± 61.7
Raclopride	2.51 ± 0.9
Eticlopride	0.25 ± 0.02

**Figure 3:**
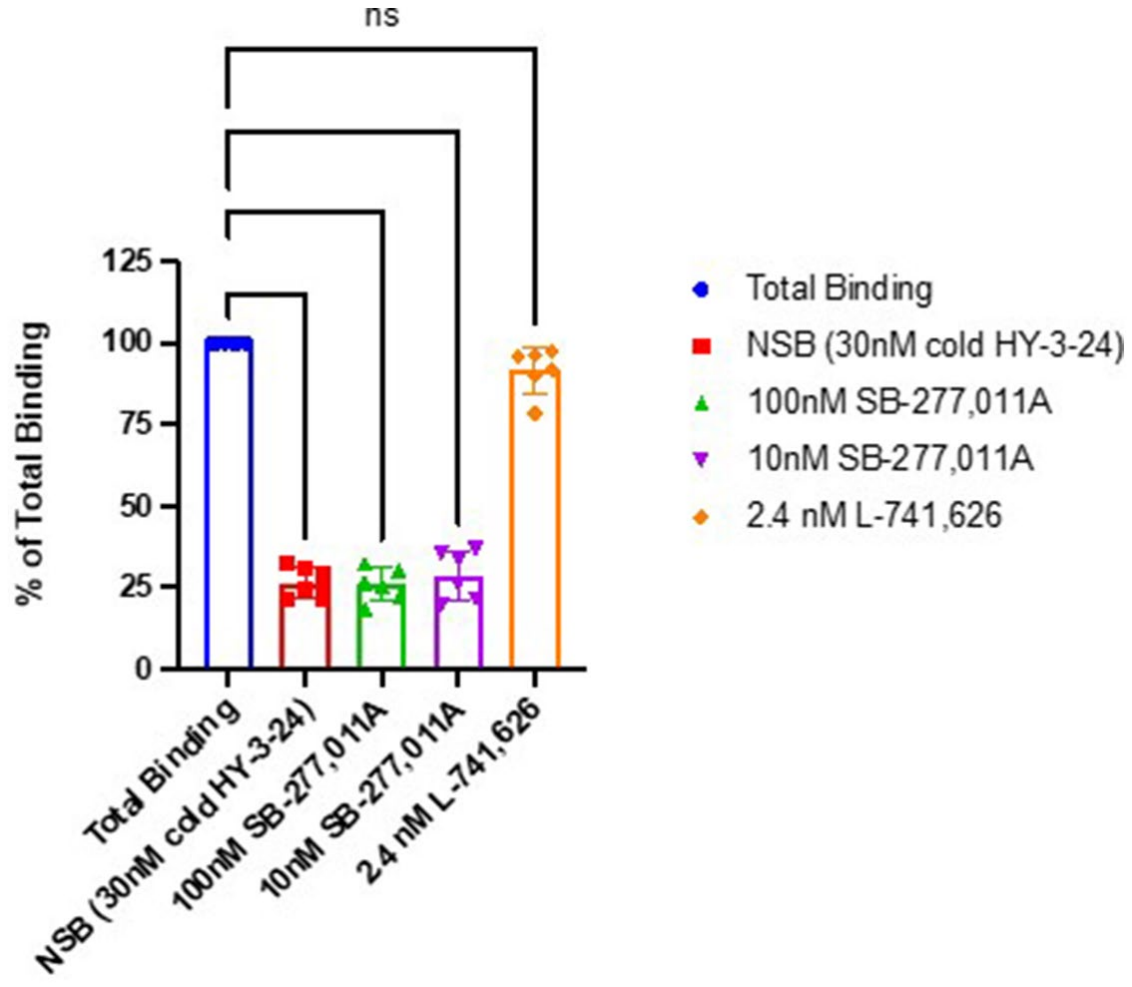
Blocking studies of [^125^I]HY-3-24 in rat striatal homogenates. Homogenized protein was incubated at 0.03 nM in the presence of 30 nM cold HY-3-24, 100 nM SB-277-011A, 10 nM SB-277-011A, or 2.4 nM L-741,626. ^****^p < 0.0001, ns, not significant (*p* > 0.05).

To confirm that the binding of [^125^I]HY-3-24 was specific for D3R, blocking studies were conducted in rat tissue homogenates using the D3-selective antagonist SB-277,011A and the D2-selective antagonist L-741,626 (Figure 3). There was complete blocking of [^125^I]HY-3-24 by SB-277,011A at 10 and 100 nM (i.e., reduced to the same level of nonspecific binding), whereas there was no displacement of [^125^I]HY-3-24 binding to rat striatal membranes at a concentration of 2.4 nM, the reported *Ki* value for displacing [^3^H]spiperone to D2 receptors (Kulagowski et al., 1996). These data confirm that [^125^I]HY-3-24 is highly selective for D3R vs. D2R.

### 3.7 Localization of [^125^I]HY-3-24 binding sites in rat and NHP brains

**Figure 4:**
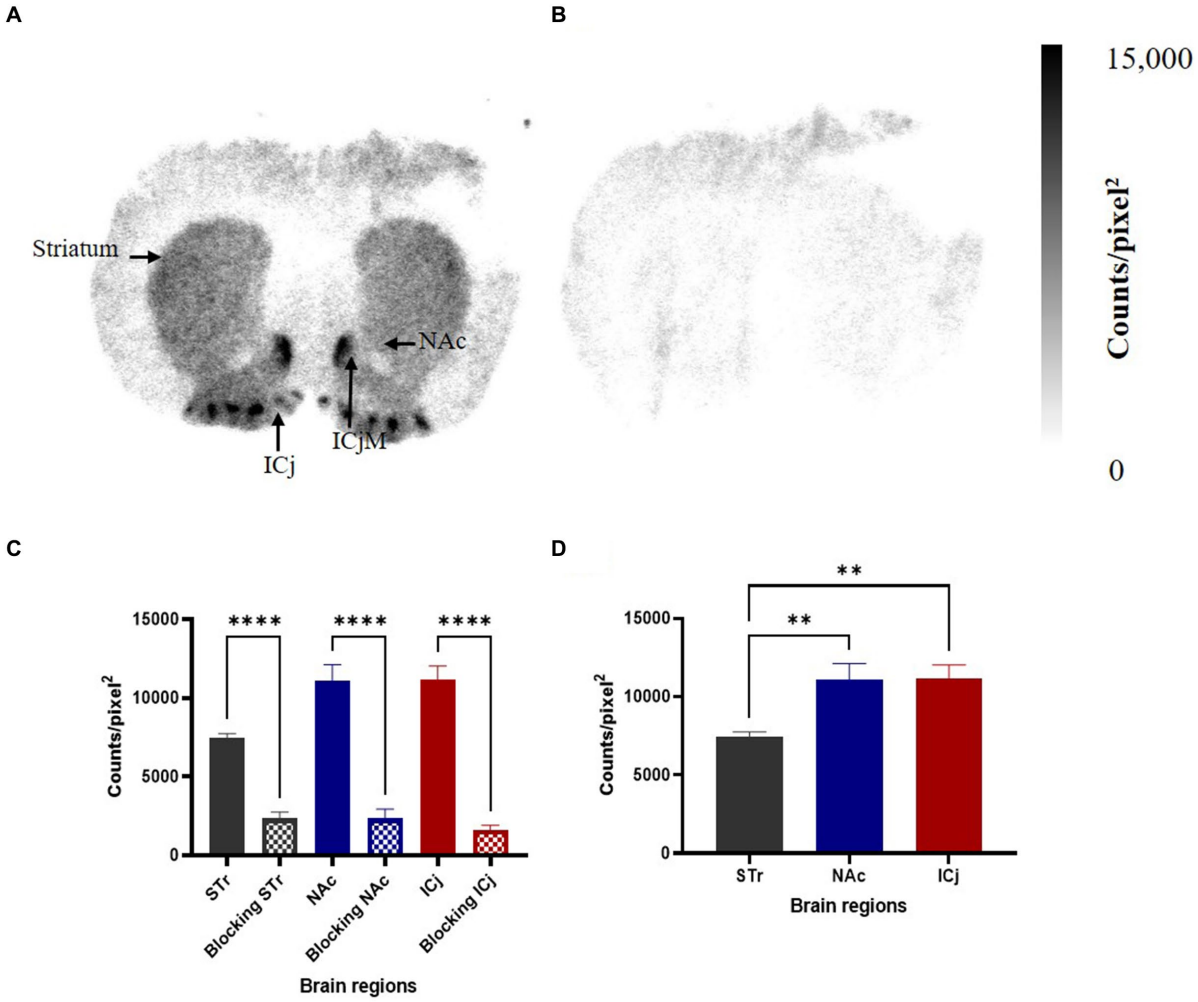
Specific binding site of [^125^I]HY-3-24 in rat brain. Sectioned brain slides (coronal direction) were incubated with ~0.3 nM of [^125^I]HY-3-24, and non-specific binding were determined by the presence of 2 μM (+)-butaclamol. The region of interest (ROI) in rat brain was selected and quantified by multi-gauge V3.0. The results (*N* = 3) are presented as a mean ± SD of three different rat brain samples. **(A)** Total binding, **(B)** non-specific binding, **(C)** quantification of results between total binding and non-specific binding (Ordinary one-way ANOVA, ^****^p < 0.0001), and **(D)** comparison between STr, NAc including ICjM, and ICj (Ordinary one-way ANOVA, ^**^p < 0.005).

The binding of [^125^I]HY-3-24 in rat brain (coronal direction) was visualized by *in vitro* autoradiography (Figure 4); non-specific binding was conducted with presence of (+)-butaclamol (Figure 4B). High binding was observed in NAc including ICjM, and ICj; a lower amount of binding of [^125^I]HY-3-24 was observed in the striatum (Figure 4A). The total binding of [^125^I]HY-3-24 (Figure 4A) and non-specific binding (Figure 4B) were quantified and compared. The total binding of [^125^I]HY-3-24 in striatum (*p* < 0.005), NAc including Islands of Calleja major (ICjM) (*p* < 0.0005), and ICj (p < 0.0005) was significantly higher than non-specific binding in the presence of (+)-butaclamol (Figure 4C). These results indicate that [^125^I]HY-3-24 is fully displaceable by (+)-butaclamol and raclopride, demonstrating that this radioligand has a low amount of non-specific binding (Supplementary Figure S7).

**Figure 5:**
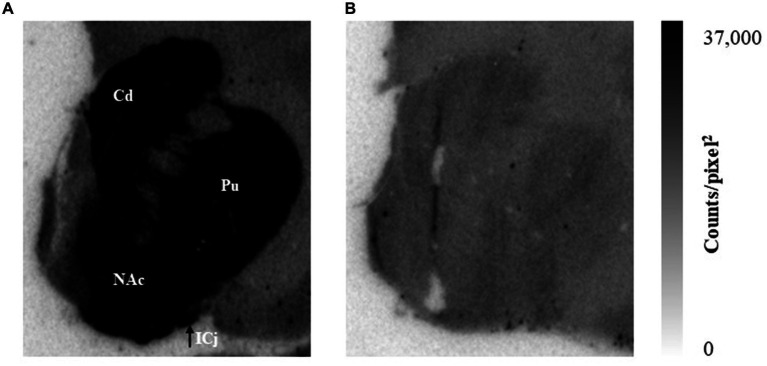
The binding site distribution of [^125^I]HY-3-24 in non-human primate brain. Slides were incubated with ~0.1 nM of [^125^I]HY-3-24. **(A)** Total binding, **(B)** non-specific binding in the presence of 2 μM (+)-butaclamol. Data were analyzed by multi-gauge V3.0.

To explore potential species differences, *in vitro* autoradiography was performed in rhesus monkey brain sections (Figure 5). The binding of [^125^I]HY-3-24 was observed on caudate nucleus (Cd), putamen (Pu), NAc, and ICj with high density (Figure 5A). As in the rat autoradiography studies, blocking with 2 μM of (+)-butaclamol demonstrated a low level of non-specific binding of the radioligand (Figure 5B).

## 4 Discussion

The dopamine D3R is considered an important CNS target since a change in density of D3 receptors is thought to be involved in a variety of neurological and psychiatric disorders. The availability of high affinity radioligands for the D3R that have low binding to D2R and D4R and can compete with endogenous dopamine would be a useful tool for studying the change in density of D3R both *in vitro* and *in vivo*. However, efforts to find highly D3-selective radioligands that can study D3 receptor expression *in vitro* and *in vivo* has been limited by their moderate to high binding to the D2R (Burris et al., 1995; Vile et al., 1995; Luedtke et al., 2000; Xu et al., 2009) or poor competition with endogenous dopamine (Schotte et al., 1996; Mach and Luedtke, 2018; Hsieh et al., 2021). Poor competition with endogenous dopamine limits the ability of a radioligand to image D3R both *in vitro* and *in vivo*. In a recent study, our group reported a structure–activity relationship (SAR) study that led to the identification of a high affinity D3-selective ligand that was potent in inhibiting the ability of dopamine binding in a *β*-arrestin recruitment assay (Kim et al., 2023). These data suggest that this ligand is capable of competing with dopamine for binding to the D3R both *in vitro* and *in vivo*. The goal of the current study was to develop a D3-selective radioligand for *in vitro* assays based on this novel scaffold. The generation of this radioligand involved a slight modification of our lead compound by replacing a Br-group with an I-group for radioiodination with I-125. In this study, ^125^I was chosen as the radionuclide because of its high specific activity relative to tritium, and its shorter half-life, which simplifies radioactive waste disposal. Therefore, [^125^I]HY-3-24 was prepared in over 99% of radiochemical purity within 70 min, and the ability of this new radioligand for targeting the D3R was evaluated in a panel of *in vitro* binding studies.

The selectivity of HY-2-34 for D3R vs. D2R and D4R was initially characterized by *in vitro* binding assays using engineered cells and the non-selective radioligand, [^125^I]IABN. The results revealed that HY-2-34 has sub-nM affinity for D3R, and significantly greater than 100-fold selectivity compared to D2R; the affinity for D4R was negligible (Table 1). The selectivity of [^125^I]HY-3-24 for D3R vs. D2R was also confirmed by conducting *in vitro* blocking studies in rat striatal homogenates using antagonists selective for the D3R (SB-277,011A) and D2R (L741,626) (Figure 3). HY-2-34 was also found to be an antagonist by using a β-arrestin recruitment assay. The high potency of HY-2-34 for inhibiting dopamine in the antagonist mode of the assay indicates that this compound has the ability to compete with dopamine for binding to the D3R, a key factor for imaging the D3R both *in vitro* and *in vivo*.

According to previous studies, ligands targeting D3 receptors often show cross-reactivity sigma receptors (Wallace and Booze, 1995; Huang et al., 2001). Therefore, sigma receptor binding studies was performed and demonstrated that HY-2-34 has a low affinity for sigma-1 and sigma-2 receptors. Likewise, the localization of [^125^I]HY-3-24 binding site *in vitro* autoradiography was indicated very low density in cortex and hippocampus noted for highly expressed regions for sigma receptors (Gundlach et al., 1986; Kim et al., 2022).

The possibility of off-target binding to other GPCRs was also a main concern for HY-2-34 because of the conformational flexibility of the molecule. Therefore, HY-2-34 was screened for binding to a panel of GPCRs such as serotonin, histamine, and opioid receptors by PDSP (Supplementary Table S1). High binding affinity was observed only at dopamine D3R, and HY-2-34 was not active or moderately active with over 100 nM affinity for the other GPCRs with the exception of dopamine D2R (*K_i_* = 43 nM) and 5-HT_2B_ receptors (*K_i_* = 38 nM). The affinity dopamine D3R was also investigated to be a *K_i_* value of 1.2 nM. The slight differences observed between PDSP and in-house methods for dopamine D2R and D3R are likely attributed to the radioligand used in the assay. While PDSD employed [^3^H]N-methylspiperone, our lab utilized ^[125^I]IABN, which displayed negligible non-specific binding (Luedtke et al., 2000; Reilly et al., 2019). Regarding the 5-HT_2B_ receptor, its localization includes the dorsal hypothalamus, frontal cortex, medial amygdala, and meninges. The low binding of [^125^I]HY-3-24 to these brain regions in the *in vitro* autoradiography studies suggests that off-target binding to 5-HT_2B_ is not likely to be a problem with this radioligand.

Since dopamine D3R was known to exist with high density in rat ventral striatum (Levesque et al., 1992; Bancroft et al., 1998), the equilibrium property of [^125^I]HY-3-24 to D3R was measured on ventral striatum homogenized by saturation experiment. Under the same conditions, Scatchard studies were conducted to measure the *K_d_* value of [^125^I]HY-3-24, which was found to be 0.34 ± 0.22 nM. Competition studies were conducted using commercially-available D3R ligands to determine the pharmacological profile of [^125^I]HY-3-24. Eticlopride had the highest affinity for competing with [^125^I]HY-3-24 to D3R (*K_i_* = 0.25 nM), which is consistent with literature value for eticlopride at the D3R (Keck et al., 2015). Raclopride has a 10-fold lower affinity for displacing [^125^I]HY-3-24 to ventral striatum followed by the D1 antagonist, SCH23390, which has a 1,000-fold lower potency in this assay. Among the tested agonists (±)-PHNO was the most potent in displacing [^125^I]HY-3-24 (*K_i_* = 1.72 nM), followed by PD128907 (*K_i_* = 7.65 nM) and Quinpirole (*K_i_* = 7.65 nM). The rank order on the *K_i_* values of the antagonists and agonists is consistent with their affinity for the D3R (Pugsley et al., 1995; Keck et al., 2015; Doot et al., 2019).

The regional distribution and density of the dopamine D3R in brain has been of great interest for many years and was initiated by the hypothesis that D3R may be important target for neurological and neuropsychiatric disorders (Sokoloff et al., 1990). Over the past 3 decades, there have been several attempts to quantify the density of the D3R in the CNS, but these studies utilized radioligands that had a suboptimal selectivity for the D3R vs. D2R (i.e., <100-fold) (Burris et al., 1995; Vile et al., 1995; Luedtke et al., 2000; Xu et al., 2009; Zhen et al., 2010). The high selectivity of HY-2-34 for D3R vs. D2R (~129-fold) suggests that a radioiodinated version of this compound may be a useful radioligand for *in vitro* binding studies and *in vitro* autoradiography studies. The low off target binding of this ligand vs. other radiolabeled D3R ligands (e.g., [^3^H]WC-10), also make this an attractive radioligand for *in vitro* autoradiography studies. The present results using [^125^I]HY-3-24 in rat brain sections confirmed that the highest density of D3R in this species was in the ICj followed by the NAc including ICjM, which is consistent with earlier observations (Schotte et al., 1996; Bancroft et al., 1998). Furthermore, our quantitative results in non-human primate brain demonstrate that the density of D3R is much higher in the striatal regions (caudate and putamen) than the striatum of rodent brain. These results are consistent with our previous studies with [^3^H]WC-10 (Xu et al., 2009, 2010). The advantage of [^125^I]HY-3-24 over [^3^H]WC-10 is that its high selectivity for the D3R avoids the uses of a duo-radioligand study with [^3^H]raclopride and a complex calculation to tease the density of the D3R from the D2R. We are currently conducting *in vitro* autoradiography studies with [^125^I]HY-3-24 in postmortem brain sections in a variety of CNS disorders.

## 5 Conclusion

In summary, our results indicate that HY-2-34 is a novel D3R selective ligand having the following features: (1) sub-nanomolar affinity (*K_i_* = 0.67 ± 0.11 nM); (2) high selectivity for D3R (>10-fold for D3R vs. D2R); (3) a high potency as a D3R antagonist (IC_50_ = 1.5 ± 0.58 nM); and (4) low affinity to other GPCRs. Furthermore, [^125^I]HY-3-24 appears to be a novel radioligand exhibiting high binding affinity and specificity at D3R: (1) *K_d_* = 0.34 ± 0.22 nM and *B*_max_ = 38.91 ± 2.39 fmol/mg protein on rat ventral striatum membrane homogenates; and (2) specific binding to NAc including ICjM and ICj in rat and NHP brain tissues. Based on all results, it is anticipated that [^125^I]HY-3-24 is promising candidate for use as the specific D3R radioligand in *in vitro* binding and *in vitro* autoradiography studies.

## Data availability statement

The original contributions presented in the study are included in the article/Supplementary material, further inquiries can be directed to the corresponding author.

## Ethics statement

The animal study was approved by Institutional Animal Care and Use Committee. The study was conducted in accordance with the local legislation and institutional requirements.

## Author contributions

JL: Writing – original draft, Methodology, Formal analysis. HK: Writing – original draft, Methodology, Investigation. PM: Writing – original draft, Methodology. AR: Writing – original draft, Methodology, Formal analysis. MT: Writing – original draft, Methodology, Formal analysis. RL: Writing – original draft, Formal analysis. RM: Writing – review & editing, Writing – original draft, Visualization, Validation, Supervision, Software, Resources, Project administration, Methodology, Investigation, Funding acquisition, Formal analysis, Data curation, Conceptualization.
