# Supplementary material for: In vitro characterization of [125I]HY-3-24, a selective ligand for the dopamine D3 receptor
Source: Front Neurosci. 2024 Apr 9;18:1380009. doi: 10.3389/fnins.2024.1380009 (PMC11036874; doi:10.3389/fnins.2024.1380009)

**Supplementary Material**

**In Vitro characterization of [^125^I]HY-3-24, a selective ligand for the dopamine D3 receptor**

**Index**

**1. hD2/D3/D4 binding assay (Figure S1)-----------------------------------------------------------------3**

**2. Sigma receptors binding assay (Figure S2)-----------------------------------------------------------4**

**3. *β*-Arrestin recruitment assay (Figure S3)-------------------------------------------------------------5**

**4. Comprehensive screening of HY-3-24 throughout various GPCRs from Psychoactive Drug Screening (PDSP) (Table S1)-------------------------------------------------------------------------------6**

**5. Representative HPLC chromatograms for labeling condition. (Figure S4)-------------------7**

**6. Analytical HPLC chromatograms for prepared [^125^I]HY-3-24 and non-radioactive standard (Figure S5)-----------------------------------------------------------------------------------------9**

**7. The equilibrium of [^125^I]HY-3-24 on rat ventral striatum membranes (Figure S6)---------10**

**8. Blocking studies with known dopaminergic agents (Figure S7)--------------------------------11**

**9. ^1^H or ^13^C NMR spectra ---------------------------------------------------------------------------------12**

**
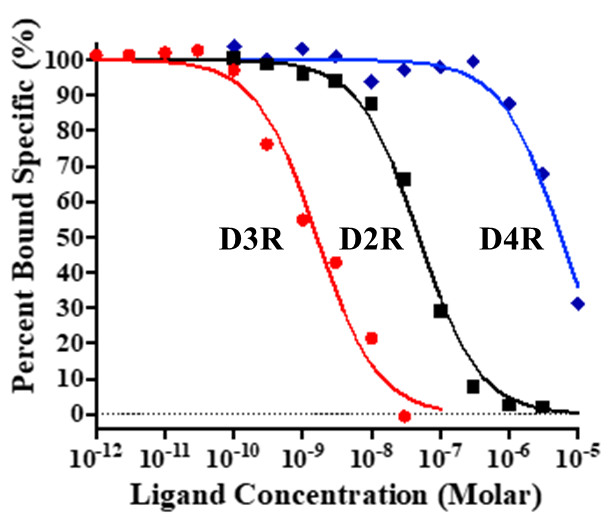
**

**Figure S1**. hD2/D3/D4R binding assay. The affinity of **HY-3-24** on dopamine receptors were obtained by receptor binding assay using [^125^I]IABN. Several concentration of **HY-3-24** (10^-10^ to 10^-5^) was prepared and incubated with [^125^I]IABN on human D2R or D3R or D4R HEK 293 cells. The graph showed represented data and the results were obtained three individual experiments. ■, D2R; ●, D3R; ♦, D4R.

**
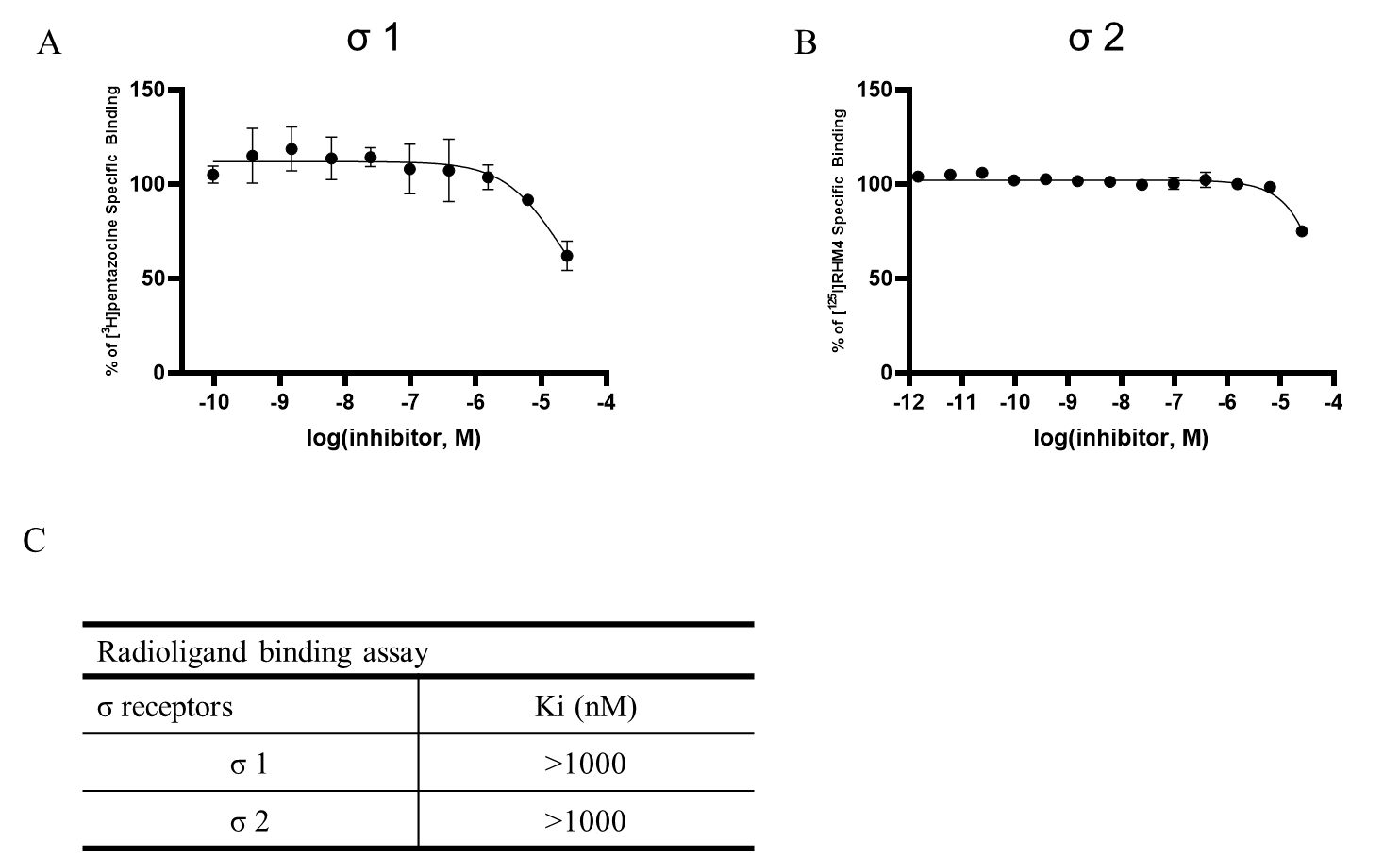
**

**Figure S2.** Sigma receptors binding assay. The affinity of **HY-3-24** on sigma (σ) receptors were confirmed by indirect binding assay using [^3^H]-(+)-pentazocine for sigma 1 (A) and [^125^I]RHM4 for sigma 2 (B) K_i_ values. Prepared **HY-3-24** (10^-11^ to 10^-5^) was incubated with guinea pig brain homogenates or rat liver homogenates. The results were obtained three individual experiments and analyzed by Prism 9.

**
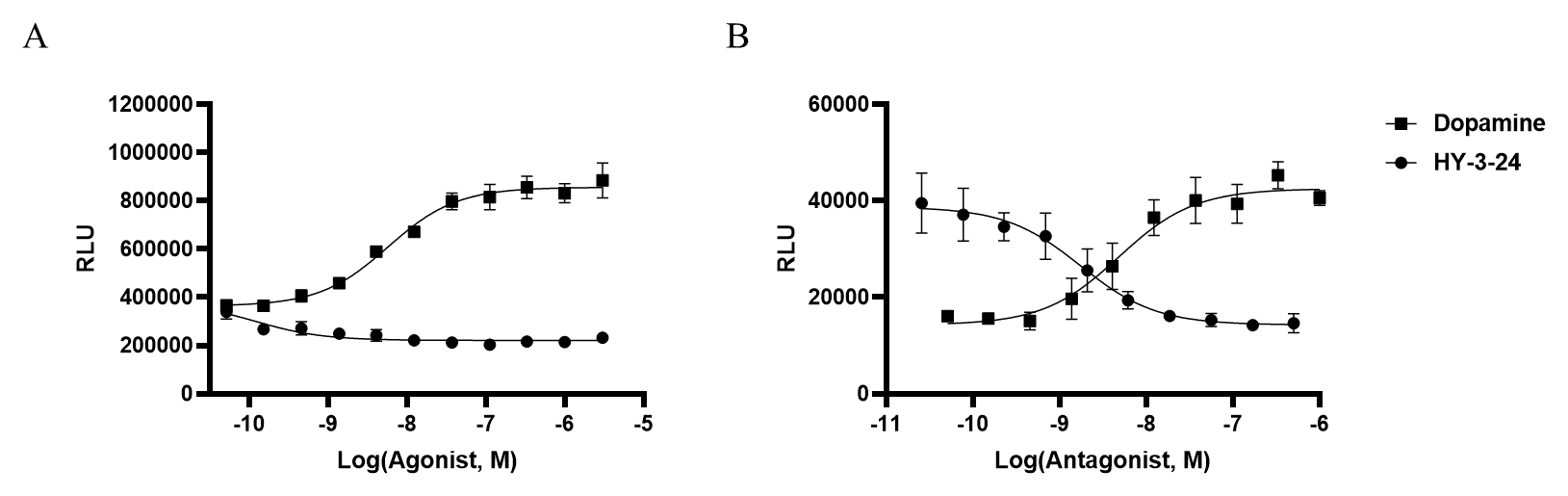
**

**Figure S3.** *β*-Arrestin recruitment assay of HY-3-24 for D3R agonist activity (EC_50_, A) and antagonist activity (IC_50_, B). **HY-3-24** was incubated on human D3R expressed CHO-K1 cells and dopamine was used as a standard. The results were obtained three individual experiments and analyzed by Prism 9. ■, Dopamine; ●, HY-3-24

**Table S1.** Comprehensive screening of HY-3-24 throughout various GPCRs from Psychoactive Drug Screening (PDSP)*^a^*

| GPCRs | | *K*i (nM) | GPCRs | | *K*i (nM) |
| --- | --- | --- | --- | --- | --- |
| dopamine | D_1_ | N.A. | Muscarinic | M_1_ | N.A. |
|  | D_2_ | 43 |  | M_2_ | 832 |
|  | D_3_ | 1.2 |  | M_3_ | 1780 |
|  | D_4_ | 1864 |  | M_4_ | 544 |
|  | D_5_ | N.A. |  | M_5_ | 118 |
| serotonin | 5-HT_1A_ | N.A. | adrenergic | alpha_1A_ | N.A. |
|  | 5-HT_1B_ | N.A. |  | alpha_1B_ | N.A. |
|  | 5-HT_1D_ | 1687 nM |  | alpha_1D_ | N.A. |
|  | 5-HT_1E_ | N.A. |  | alpha_2A_ | 2153 |
|  | 5-HT_2A_ | 310 |  | alpha_2B_ | 1069 |
|  | 5-HT_2B_ | 38 |  | alpha_2C_ | 1898 |
|  | 5-HT_2C_ | 613 |  | beta_1_ | N.A. |
|  | 5-HT_3_ | 904 |  | beta_2_ | 5829 |
|  | 5-HT_5A_ | N.A. |  | beta_3_ | N.A. |
|  | 5-HT_6_ | 1038 | sigma | sigma_1_ | 1955 |
|  | 5-HT_7A_ | N.A. |  | sigma_2_ | 2312 |
| histamine | H_1_ | 244 | benzodiazepine rat brain site | | N.A. |
|  | H_2_ | N.A. | ɤ-aminobutyric acid type A | | 1116 |
|  | H_3_ | N.A. | dopamine active transporter | | 1126 |
|  | H_4_ | N.A. | norepinephrine transporter | | 5953 |
| opioid | MOR | N.A. | serotonin transporter | | 677 |
|  | KOR | 1546 | benzodiazepine receptor | | 5644 |
|  | DOR | N.A. |  | |  |

**^a^HY-3-24** was converted to HCl salt prior to tests. ^b^% of inhibition was measured using 10 µM of **HY-3-24**. ^c^N.A; not active, less than 50% inhibition in primary binding assays at 10 µM.

**A. 7 at room temperature incubation**


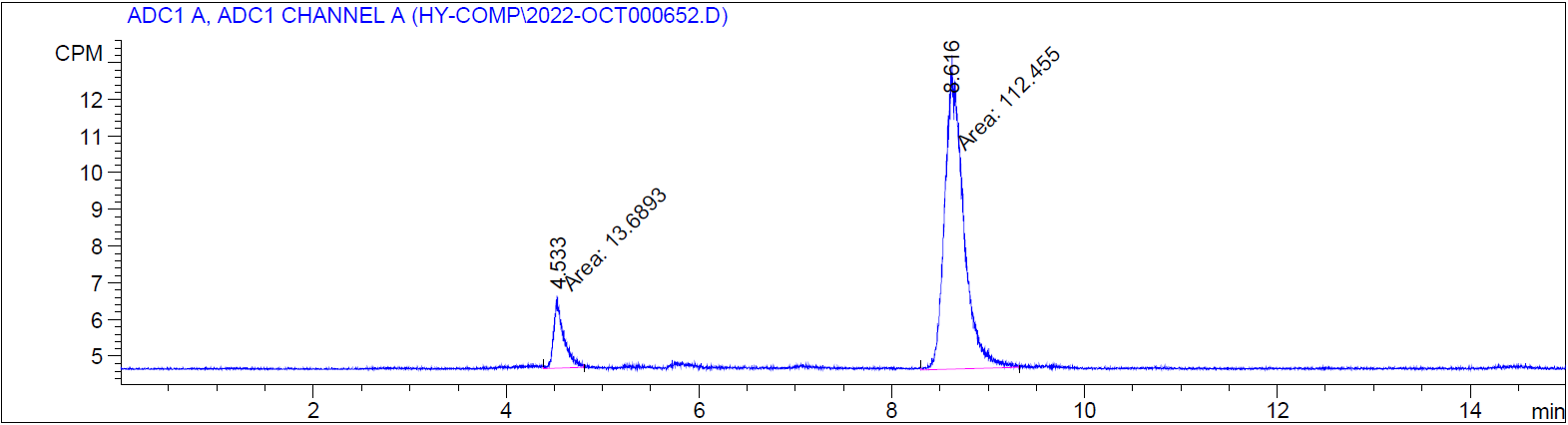


**B. 7 at 100 °C heating**


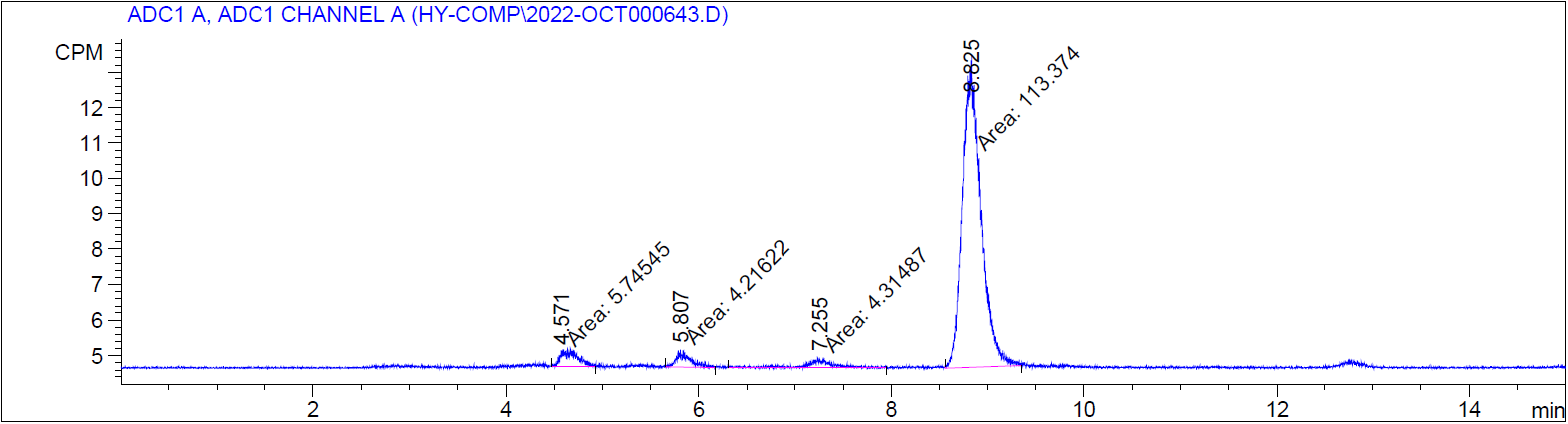


**C. 8 at room temperature incubation**


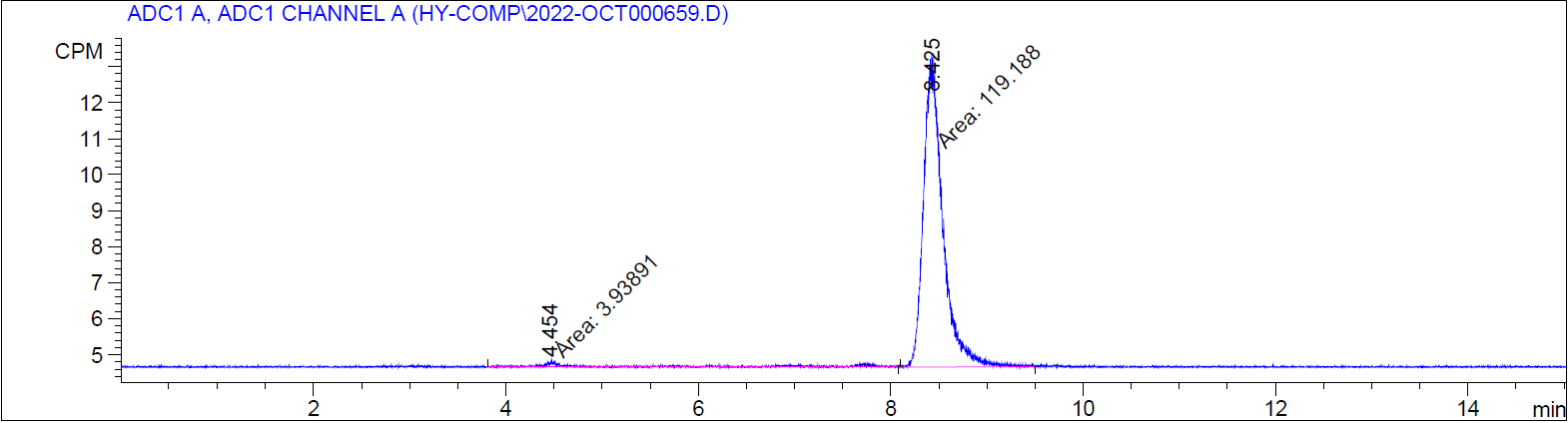


**D. 8 at 100 °C heating**


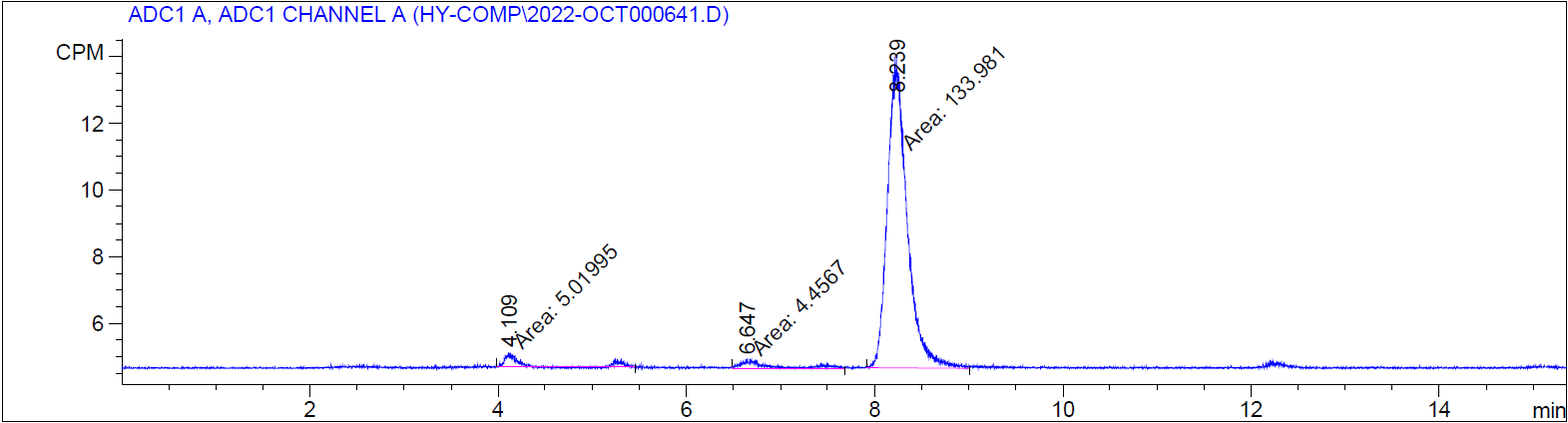


**Figure S4.** Representative HPLC chromatograms for labeling condition; stationary phase: Luna® 5 µm C18 100 Å, 10 × 250 mm, mobile phase: 68% 0.1 M ammonium acetate buffer in MeCN, wavelength 254 nm, flow rate: 4 mL/min.


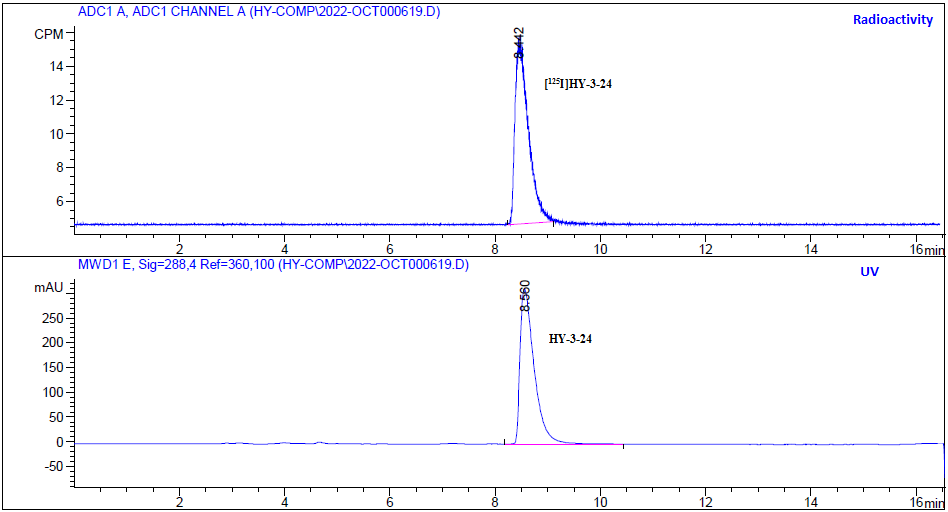


**Figure S5.** Analytical HPLC chromatograms for prepared **[^125^I]HY-3-24** and non-radioactive standard**;** stationary phase: Luna® 5 µm C18 100 Å, 10 × 250 mm, mobile phase: 68% 0.1 M ammonium acetate buffer in MeCN, wavelength 254 nm, flow rate: 4 mL/min.


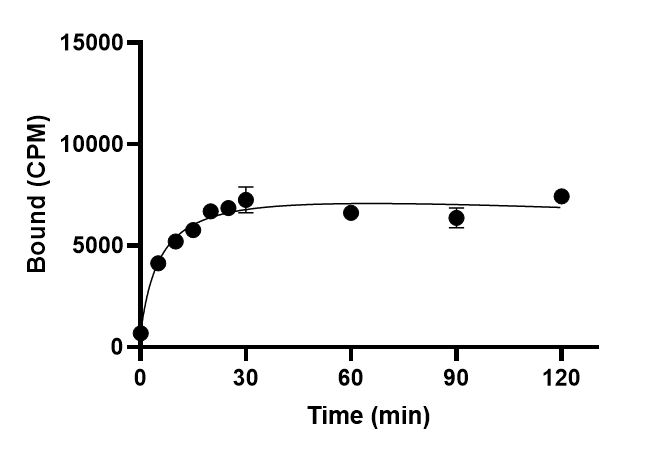


**Figure S6**. The equilibrium of **[^125^I]HY-3-24** on rat ventral striatum membranes. **[^125^I]HY-3-24** was incubated on rat ventral striatum membranes to confirm saturation time. The result was checked following time point (0, 5, 10, 15, 20, 25, 30, 60, and 120 min) by triplet.

**
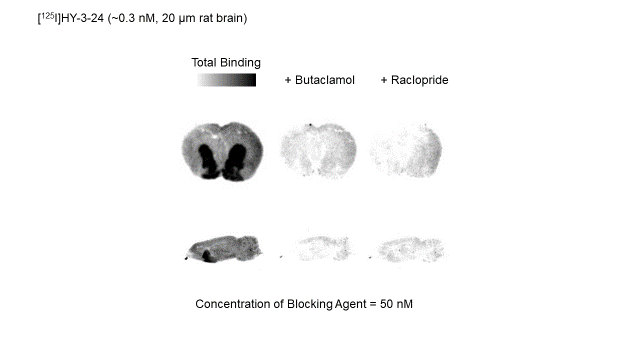
**

**Figure S7. Blocking studies with known dopaminergic agents.** The figure displays three sets of autoradiography images, each featuring a top image (coronal view) and a bottom image (sagittal view). These images were obtained for blocking studies using two different dopaminergic blocking agents: 50 nM of (+)-butaclamol and raclopride. The total binding of [^125^I]HY-3-24 was measured at a concentration of approximately 0.3 nM in 20 µm rat brain slices.

**^1^H or ^13^C NMR spectra**


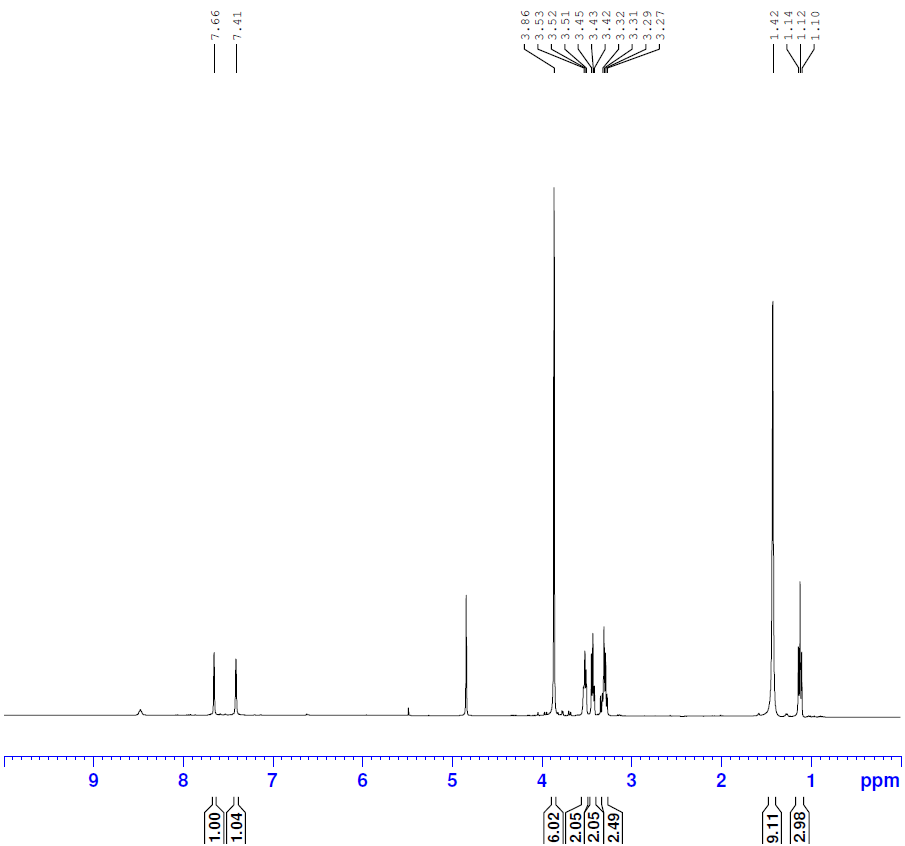


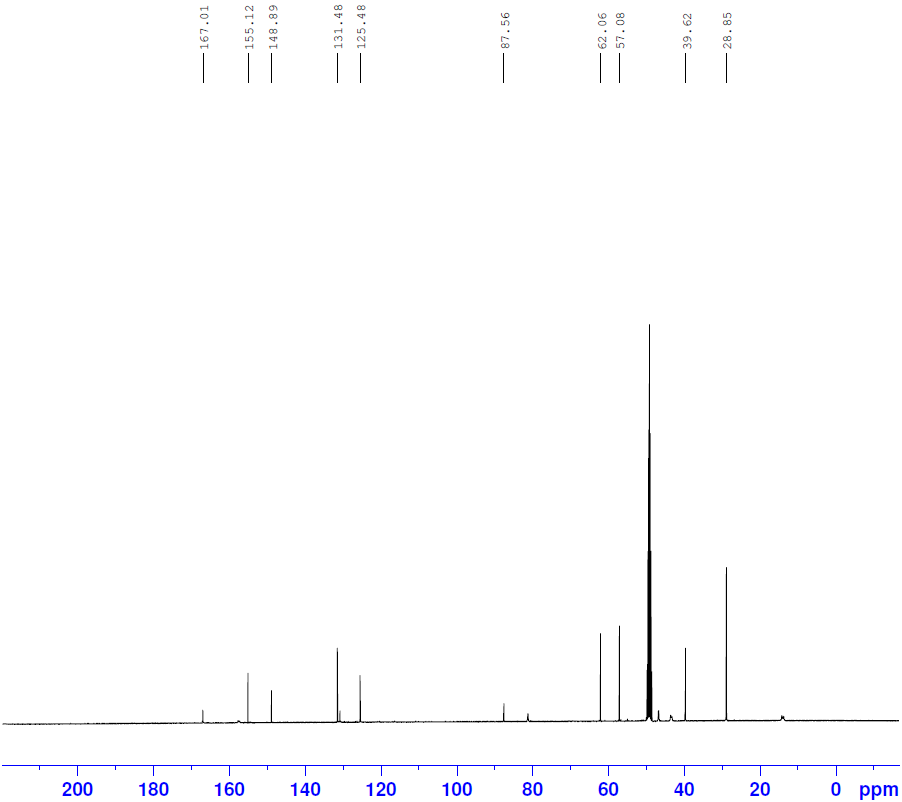


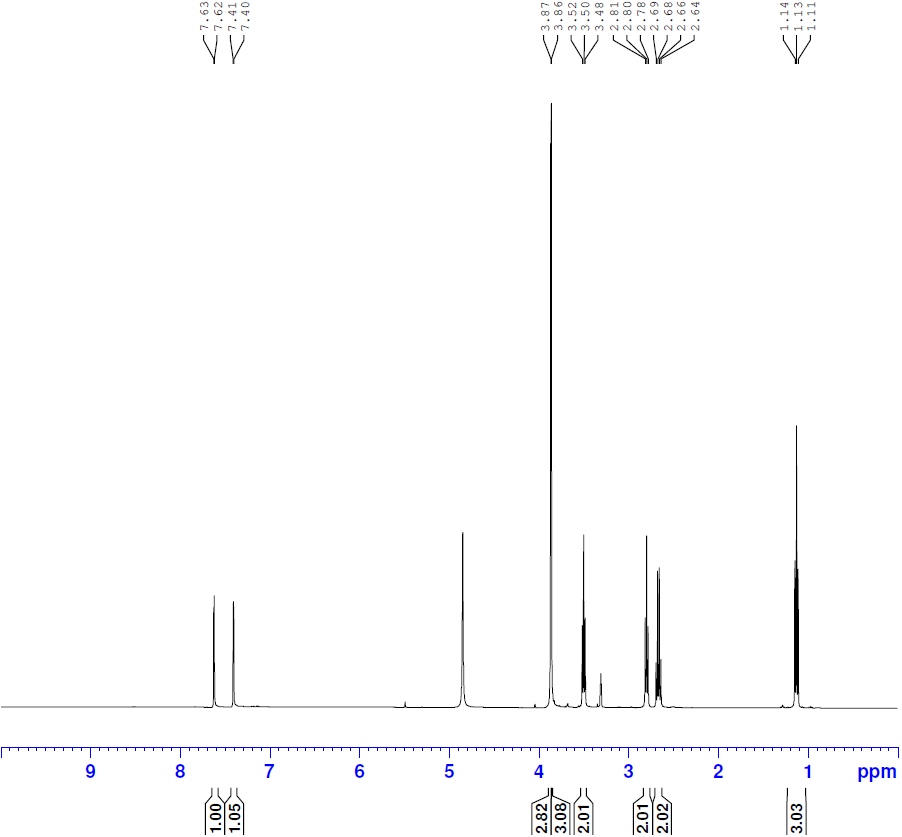


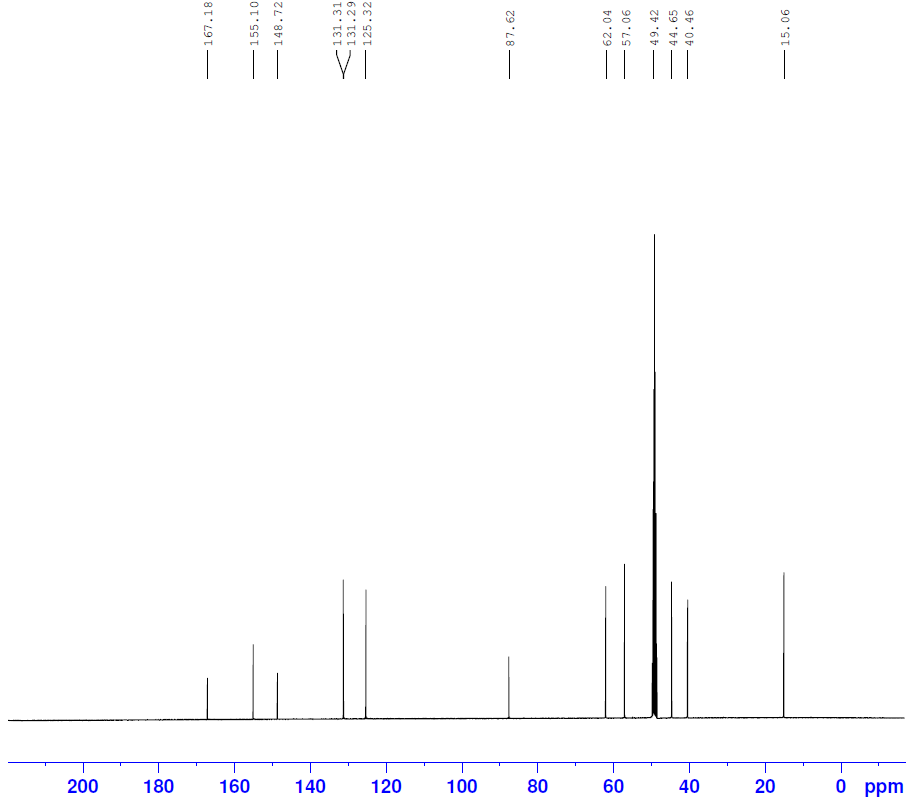


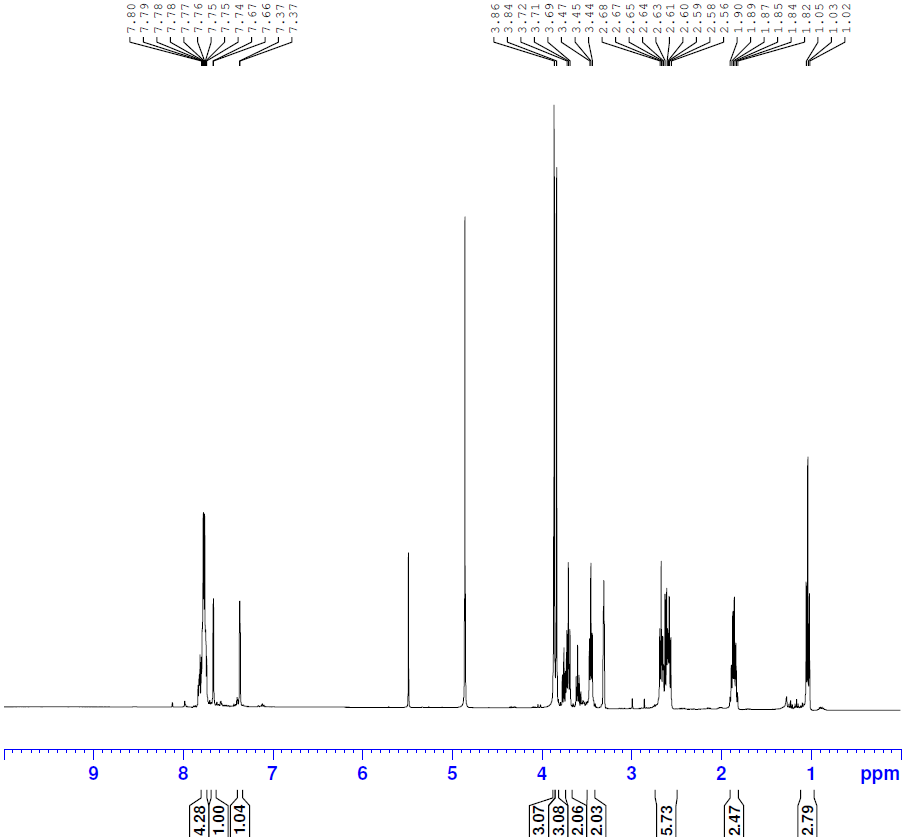


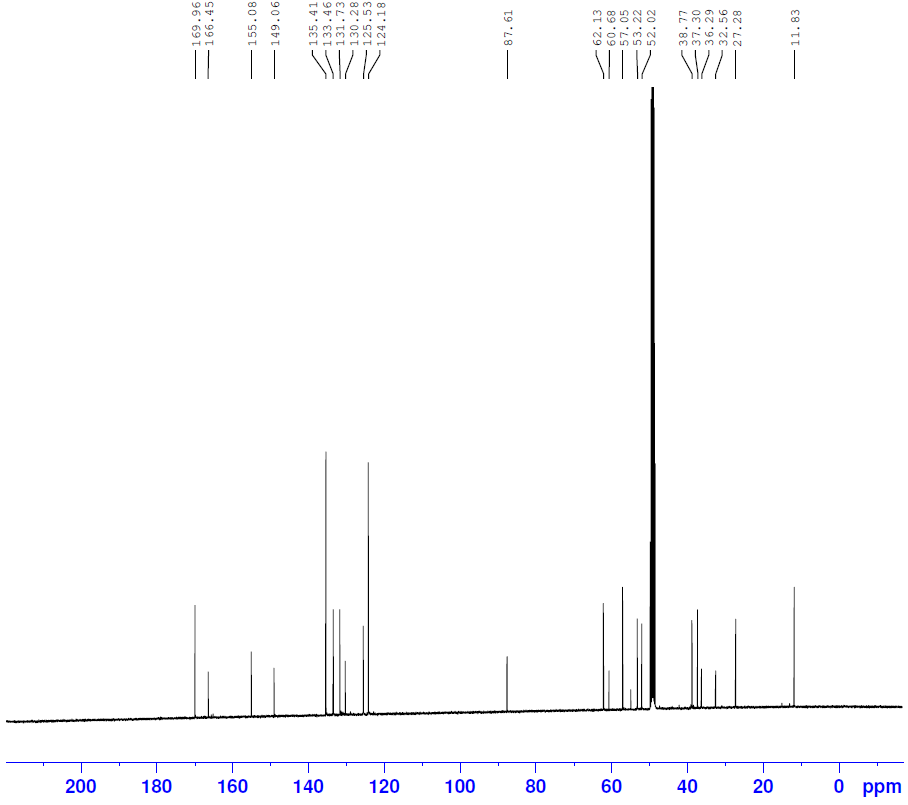


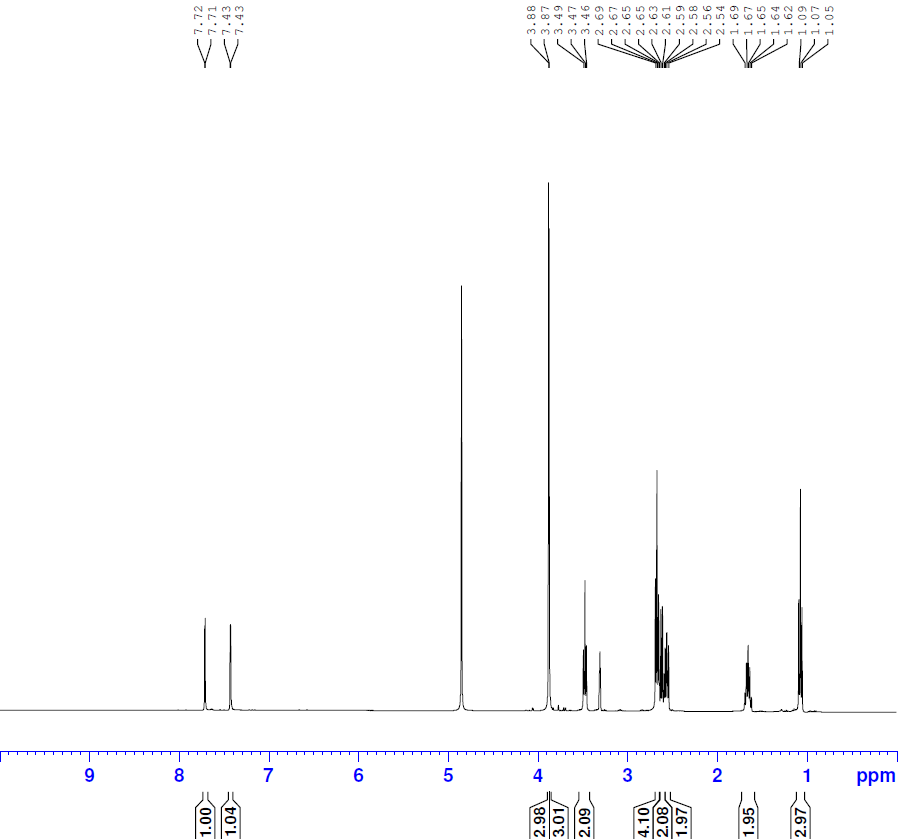


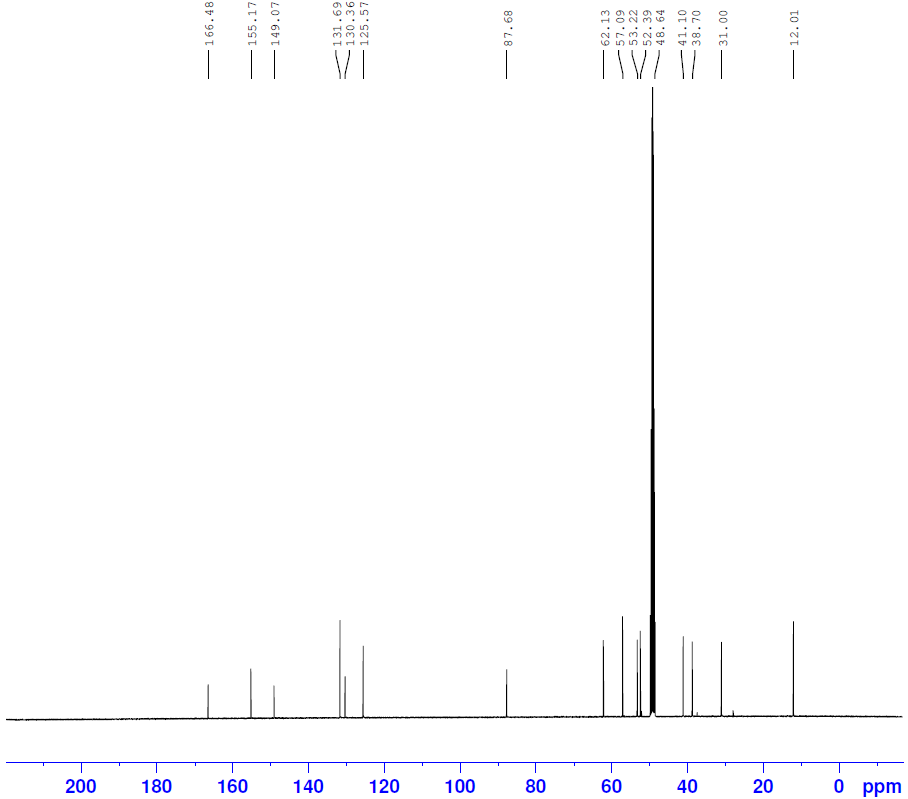


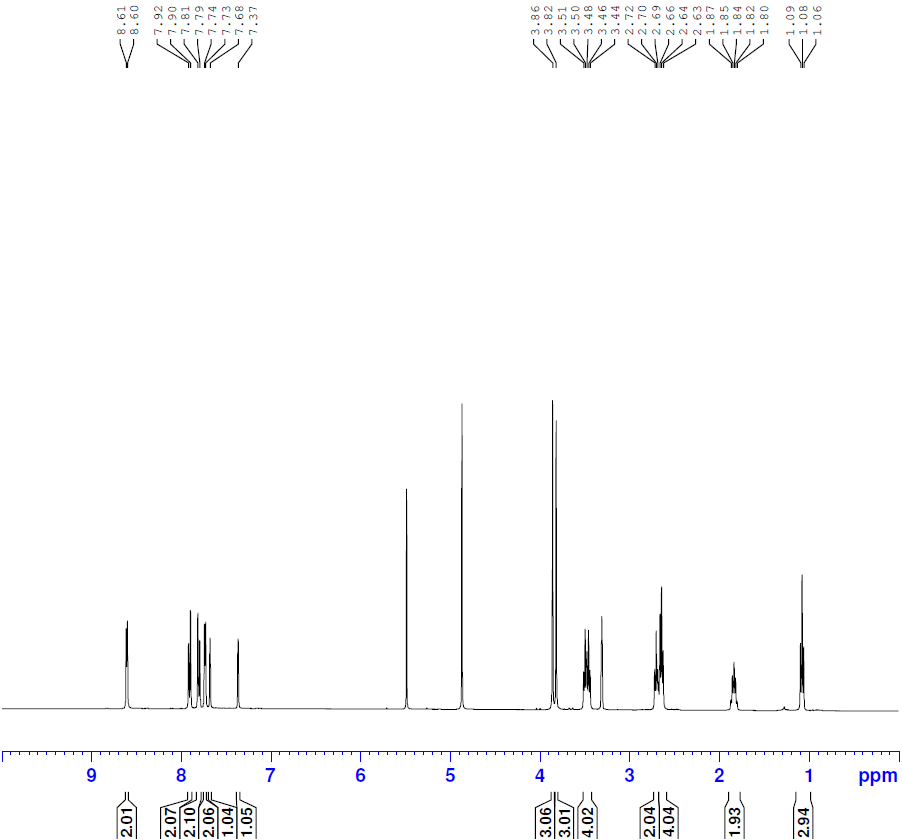


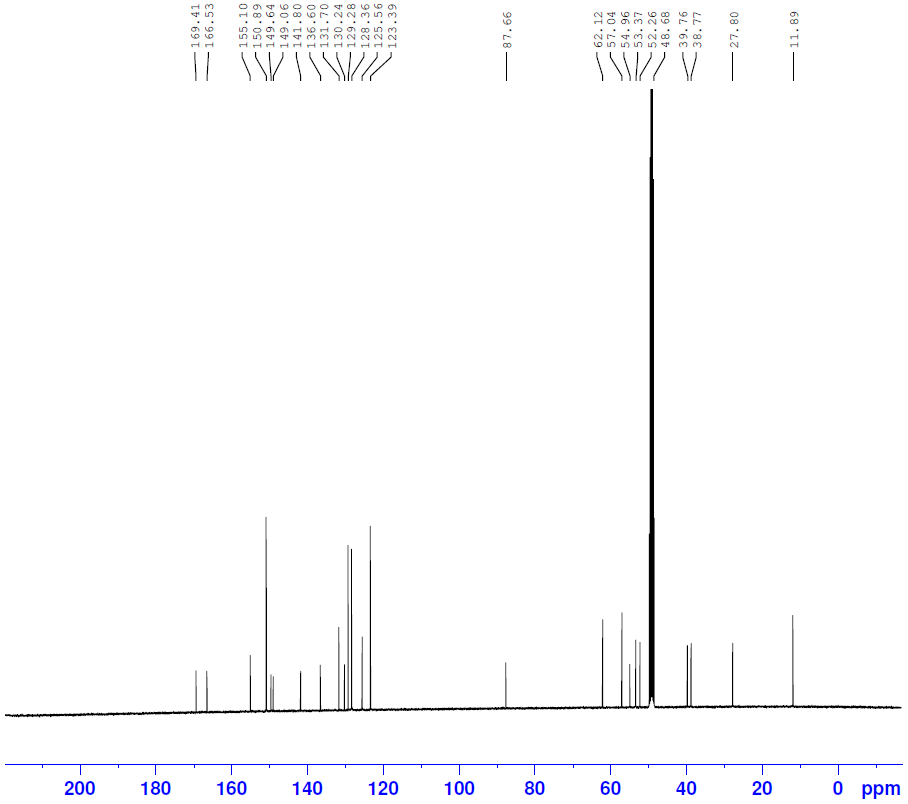


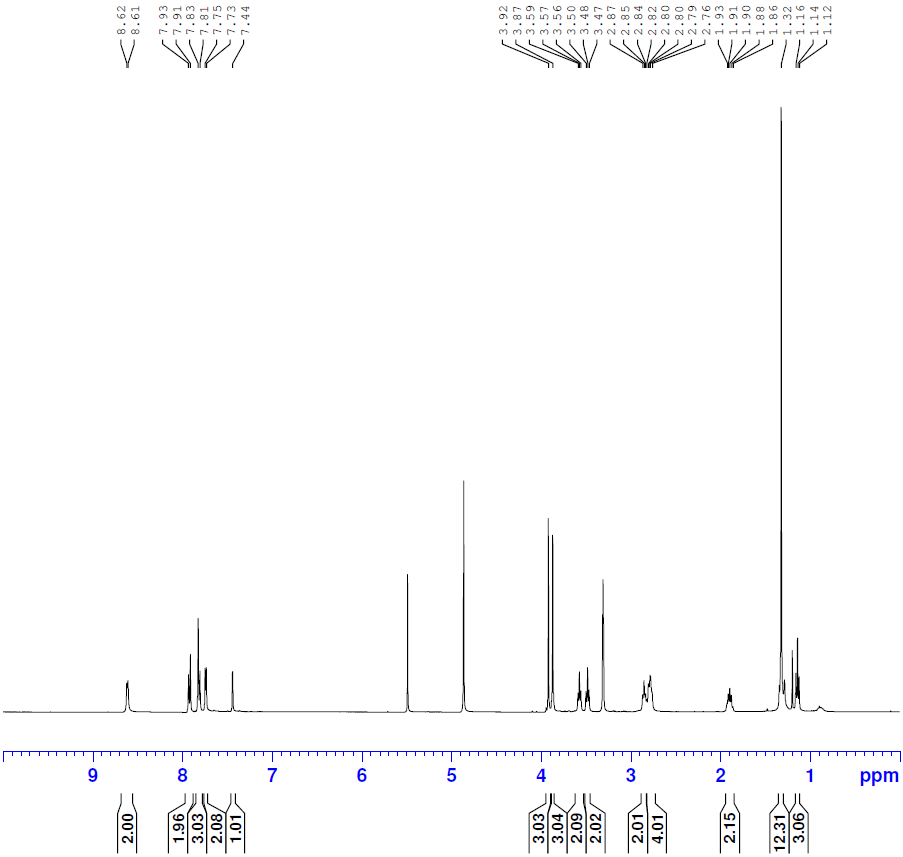


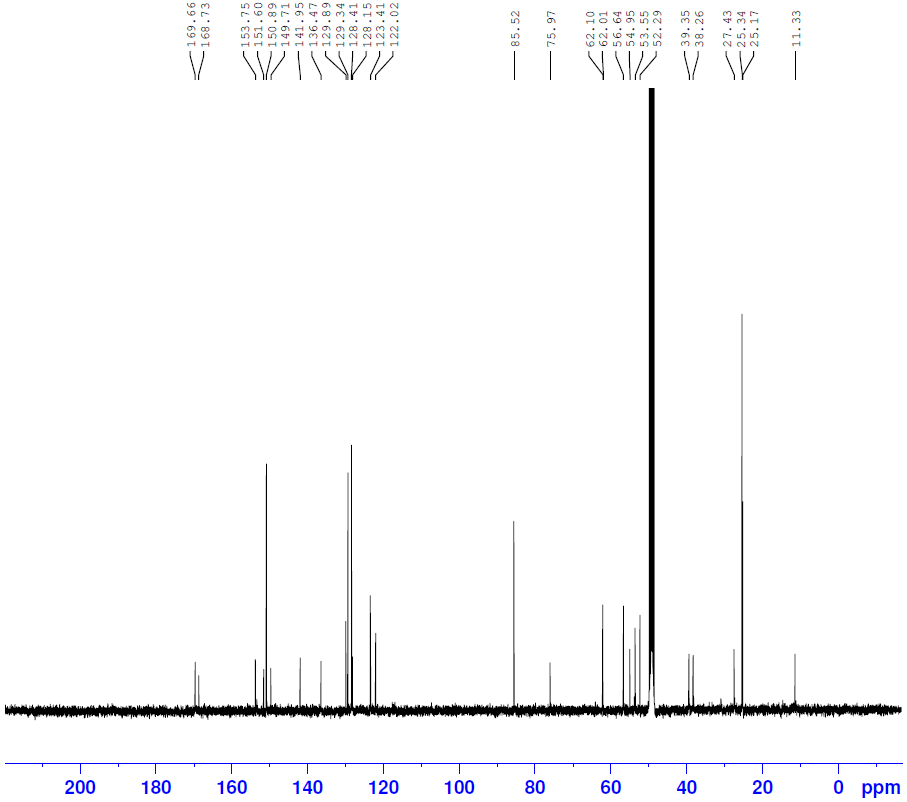


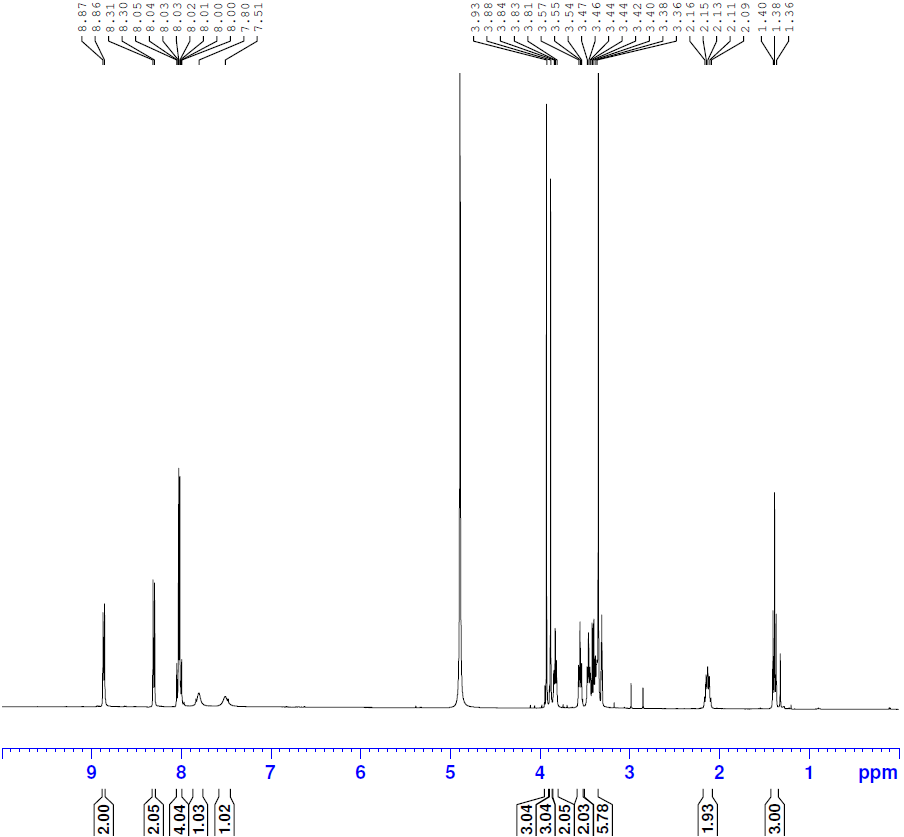


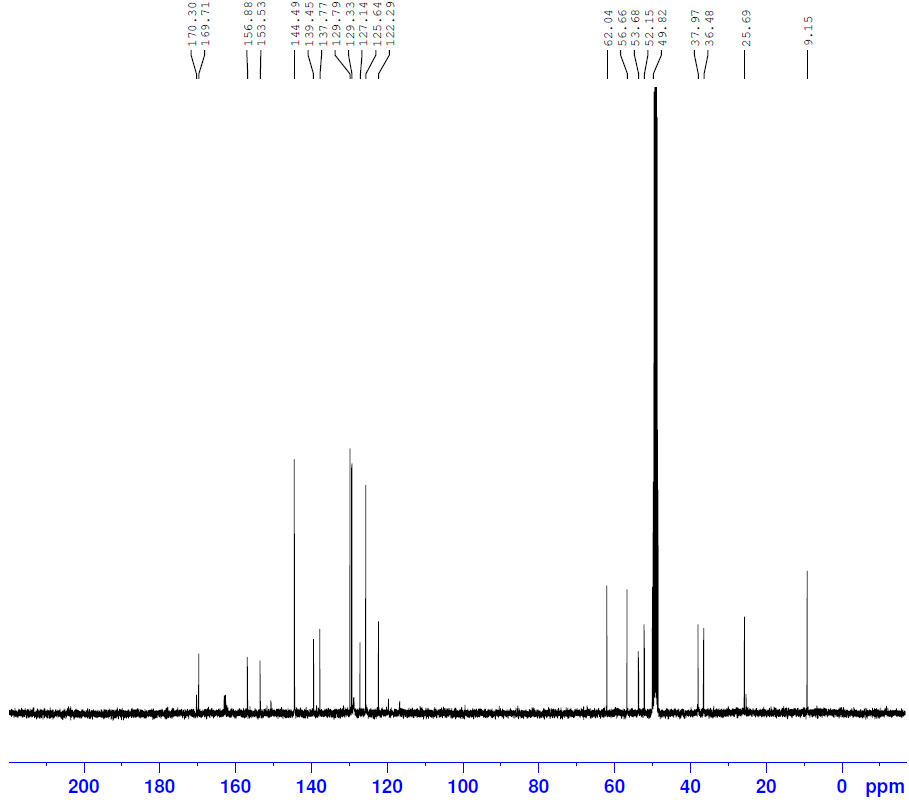

Supplement: Supplementary file 1 [file Data_Sheet_1.docx]
